# From Hemostasis to Angiogenesis: A Self-Healing Hydrogel Loaded with Copper Sulfide-Based Nanoenzyme for Whole-Process Management of Diabetic Wounds

**DOI:** 10.34133/bmr.0208

**Published:** 2025-05-23

**Authors:** Chuankai Zhang, Peirong Zhou, Shoucheng Li, Xuancheng Zhang, Zhaoxin Xia, Zihan Rao, Xuemin Ma, Yajuan Hu, Yongcen Chen, Junliang Chen, Yun He, Gang Tao, Rui Cai

**Affiliations:** ^1^ Luzhou Key Laboratory of Oral & Maxillofacial Reconstruction and Regeneration, The Affiliated Stomatological Hospital, Southwest Medical University, Luzhou 646000, China.; ^2^ Department of Oral and Maxillofacial Surgery, The Affiliated Stomatological Hospital, Southwest Medical University, Luzhou 646000, China.; ^3^ Institute of Stomatology, Southwest Medical University, Luzhou 646000, China.; ^4^ Department of General Dentistry, The Affiliated Hospital, Southwest Medical University, Luzhou 646000, China.; ^5^ Department of Oral and Maxillofacial Surgery, The Affiliated Hospital, Southwest Medical University, Luzhou 646000, China.

## Abstract

Diabetic wounds pose considerable healing challenges due to factors such as impaired angiogenesis, persistent inflammation, elevated levels of reactive oxygen species, and bacterial infections. In this study, we synthesized copper sulfide nanoparticles (NPs) using sericin as a biotemplate and functionalized them with tannic acid–Fe (TA-Fe) metal–phenolic network coatings to create CuS-based nanoenzymes (CuS-Se@TA-Fe NPs). These NPs were integrated into a composite hydrogel formed from polyvinyl alcohol, carboxymethyl chitosan, and borax. The hydrogen bonding between polyvinyl alcohol and carboxymethyl chitosan, combined with the borate ester bonds from borax and the electrostatic interactions with CuS-Se@TA-Fe NPs, resulted in a hydrogel with remarkable adhesion, self-healing capabilities, and shape retention (PCCuT hydrogel). Additionally, the PCCuT hydrogel demonstrated superoxide dismutase and catalase mimetic activities to eliminate excess free radicals, along with excellent photothermal conversion and antimicrobial properties due to the photothermal effect. Both in vitro and in vivo investigations indicated that the PCCuT hydrogel could enhance angiogenesis and promote the transformation of macrophages into the M2 anti-inflammatory phenotype. Notably, in a rat model of diabetic wound infection, the hydrogel exhibited substantial wound-healing benefits. In summary, the PCCuT hydrogel holds promise for advancing the treatment of diabetic wounds complicated by infection.

## Introduction

According to the latest report from the World Health Organization, the worldwide diabetes population reached 529 million in 2021 [1]. This chronic, noncommunicable disease has emerged as a remarkable social and economic challenge. Diabetic patients often experience complications with wound healing, such as diabetic foot ulcers, primarily due to the sustained high blood glucose levels that promote apoptosis in vascular endothelial cells, thereby impairing angiogenesis [2]. Additionally, persistent inflammation at the wound site complicates the healing process [3]. Macrophages, key players in the innate immune system, are crucial for modulating the inflammatory response in diabetic wounds. The initial pro-inflammatory phase is predominantly influenced by classically activated macrophages (M1), while the later phase is characterized by alternatively activated macrophages (M2), which help to reduce inflammation and support tissue repair [4]. However, prolonged activation of M1-type macrophages results in the overproduction of pro-inflammatory cytokines, including interleukin-6 (IL-6) and excessive reactive oxygen species (ROS), all contributing to a heightened inflammatory response that hinders healing [5,6]. Furthermore, diabetic wounds are often complicated by bacterial infections, which significantly affect their recovery and potentially lead to severe complications [7]. As a result, the development of multifunctional dressings that incorporate pro-angiogenic, ROS scavenging, immunomodulatory, and antimicrobial properties holds considerable promise for enhancing the treatment of diabetic wounds.

Nanoenzymes represent a unique category of mimetic enzymes that combine the properties and catalytic functions of nanomaterials [8]. Unlike natural enzymes, nanoenzymes offer advantages such as enhanced catalytic efficiency, stability, cost-effectiveness, and scalability, making them promising candidates for treating conditions like diabetic wounds, arthritis, and periodontitis [9]. For instance, nanoenzymes with superoxide dismutase (SOD)-like activity facilitate the conversion of superoxide anions (O_2_^•−^) into hydrogen peroxide (H_2_O_2_), which is crucial for combating microbial infections [10]. Conversely, those exhibiting catalase (CAT)-like activity can decompose H_2_O_2_ into O_2_, thereby reducing oxidative stress and promoting healing in diabetic wounds [11]. Copper sulfide (CuS), a prevalent nanoenzyme, is effective in bacterial elimination through localized H_2_O_2_ release during photothermal therapy, attributable to its SOD-like activity [12]. Additionally, the release of copper ions promotes endothelial cell migration and acts as a promoter for angiogenesis [13]. Tannic acid (TA), a naturally derived polyphenol from plants that is recognized for its antimicrobial and anti-inflammatory properties, is Food and Drug Administration approved for various applications [14]. The interaction of TA with Fe(III) results in the formation of a tannic acid–Fe (TA-Fe) metal–phenolic network (MPN) with CAT-like properties, enabling the conversion of excess H_2_O_2_ into O_2_, which alleviates hypoxic conditions at the wound site [15]. Moreover, TA exhibits a distinctive anti-inflammatory effect, aiding macrophage polarization and modulating the inflammatory reaction [16]. Thus, we propose that TA-Fe-modified CuS can catalyze a cascade of reactions at diabetic wound sites, facilitating the healing process.

Currently, the predominant approach for managing infected wounds involves antibiotic treatment; however, the misuse of drugs can result in the appearance of resistant bacteria, which can seriously affect the effectiveness of treatment. Photothermal therapy, which has received widespread attention in recent years, represents a new type of antimicrobial treatment that does not produce drug-resistant bacteria [17]. Photothermal agents are able to convert near-infrared (NIR) light into heat energy, thereby causing thermal damage to bacteria and eliminating them [18]. CuS has been extensively studied as an excellent photothermal agent because of its high photothermal conversion efficiency [19]. However, the traditional methods for the preparation of CuS nanoparticles (NPs) have harsh requirements on the reaction conditions, and the cumbersome process affects the performance of the nanomaterials [20]. In recent years, the emergence of the use of bovine serum protein as a template for the preparation of CuS NPs has suffered from high prices [21]. Sericin is a natural protein in silkworm cocoons with excellent biocompatibility and biodegradability [22]. Sericin molecules possess a loose, disordered spatial structure, with multiple amino acid residues containing long side chains and polar hydrophilic groups on the surface of the polypeptide chains. These residues can coordinate with copper ions, thereby forming copper sulfide NPs at the nanoscale. In addition, the availability and stable physicochemical properties of sericin make it an ideal material for biomedical applications [23]. Here, we obtained CuS–sericin (CuS-Se) NPs with high photothermal conversion, biocompatibility, and low cost by in situ complexation of sericin. Then, the TA-Fe MPN was incorporated, which can be used as an active coating for photothermal antimicrobial and antioxidant agents to achieve more effective bactericidal properties.

Hydrogels are promising for medical applications because of their wet-soft nature and unique bionic properties [24]. For more complex diabetic wounds, hydrogel dressings need to be biocompatible and have excellent biologic properties, as well as shape adaptation and adhesion to irregular wound tissue. Therefore, we need a multifunctional hydrogel with exceptional physical properties that can also destroy bacterial infections, inhibit inflammatory responses, remove ROS, and promote angiogenesis. Polyvinyl alcohol (PVA) is a safe polymer organic substance widely used in the medical field because it has no adverse effects on the human body. Carboxymethyl chitosan (CMCS) is a derivative of chitosan with excellent biocompatibility. In addition, CMCS has excellent hemostatic properties [25]. By mixing PVA, CMCS, and borax, dynamic borate ester and hydrogen bonds can be formed between them to obtain hydrogels with excellent self-healing and adhesion performance. Loading CuS-Se@TA-Fe NPs into the above hydrogels will result in multifunctional hydrogel dressings with photothermal antimicrobial, ROS scavenging, immunomodulatory, angiogenic, and hemostatic properties.

**Fig. 1.**
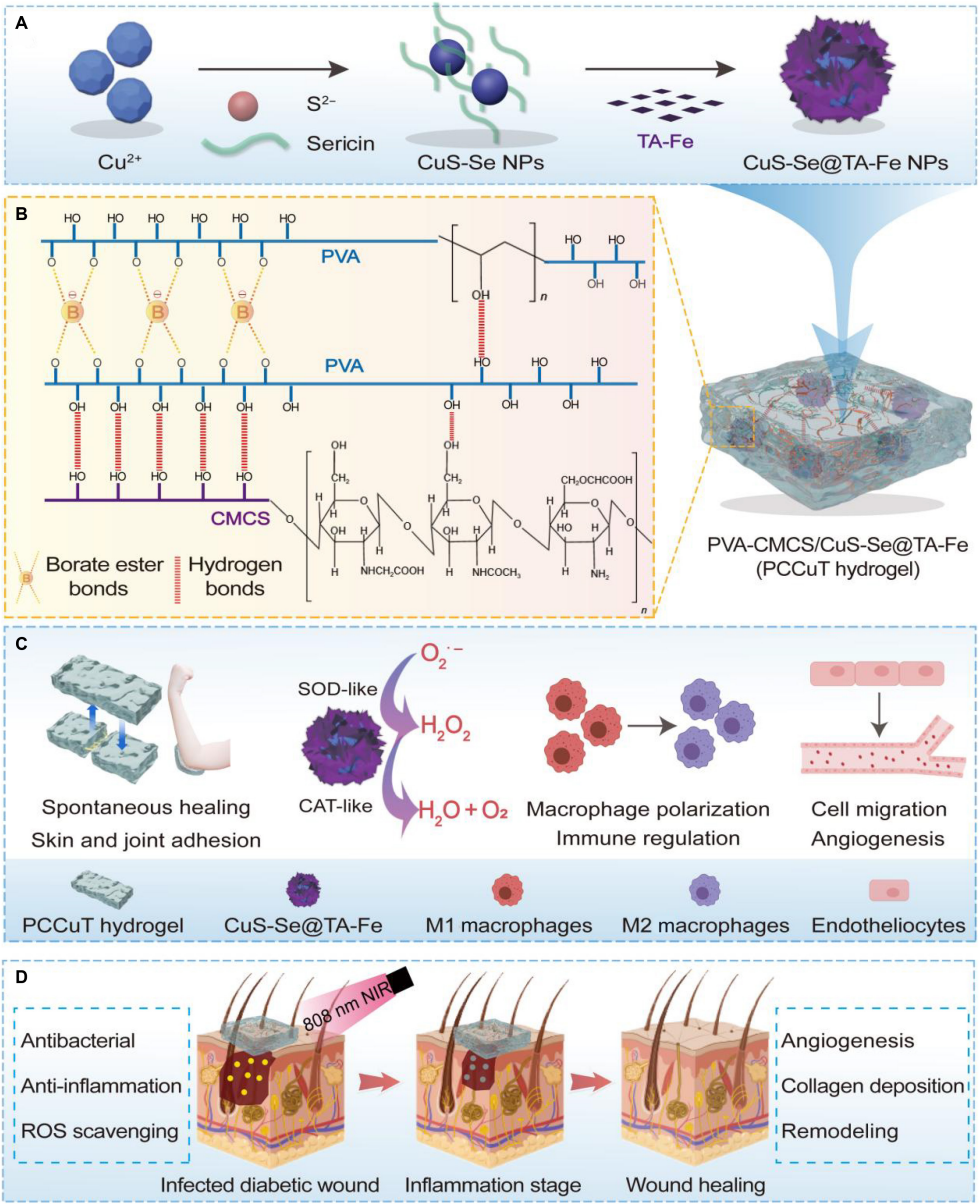
Schematic representation of the synthesis of the PCCuT hydrogel and its treatment of diabetic infected wounds. (A) Synthesis of CuS–sericin (CuS-Se) nanoparticles (NPs). (B) Diagram of the structural network of the hydrogel. (C) Schematic representation of the adhesion and self-healing performance of the hydrogel, the activity of the nanoenzymes, and the promotion of vasculogenesis as well as macrophage polarization. (D) Mechanism of the PCCuT hydrogel promoting wound healing in all stages of diabetes. TA-Fe, tannic acid–Fe; PVA, polyvinyl alcohol; CMCS, carboxymethyl chitosan; SOD, superoxide dismutase; CAT, catalase; ROS, reactive oxygen species.

In this study, we fabricated CuS-Se NPs using sericin as a biological template and functionalized TA-Fe nanocoatings on the surface, which endowed the CuS-Se NPs with anti-inflammatory and antioxidant capabilities (Fig. 1A). Then, the CuS-Se@TA-Fe NPs were further loaded into a PVA/CMCS composite hydrogel to obtain a trauma dressing with exceptional self-healing properties as well as high adhesion to biotic and abiotic tissues (Fig. 1B and C). The cytocompatibility of the composite hydrogel was estimated using human umbilical vein endothelial cells (HUVECs) and RAW264.7 cells, indicating excellent biocompatibility. 1,1-Diphenyl-2-pyridylhydrazine (DPPH) and 2,2′-azobis(3-ethylbenzothiazoline-6-sulfonic acid) (ABTS) radical scavenging tests demonstrated that the composite hydrogel exhibits remarkable antioxidant capacity. Scratch and tube-forming experiments proved the ability of the composite hydrogel to promote HUVEC migration and angiogenesis. In addition, the immunomodulatory capacity of the composite hydrogel was demonstrated in RAW264.7 cells. In vitro antimicrobial experiments demonstrated that the composite hydrogel has excellent antimicrobial ability under NIR light irradiation. Furthermore, animal model experiments showed that the hydrogel has an important effect on promoting the healing of diabetic infected wounds (Fig. 1D). In summary, the composite hydrogel holds great potential for application in the treatment of diabetic infected wounds.

## Materials and Methods

### Synthesis and characterization of CuS-Se@TA-Fe NPs

Initially, cocoons were boiled at 0.1 MPa and 121 °C for 30 min to extract sericin after removing the silk fibroin. Next, to synthesize nanoscale CuS, a solution containing [Cu(NH_3_)_4_]^2+^ was prepared by mixing 0.1 M CuSO_4_ with NH_3_·H_2_O. This solution was then diluted with 10 ml of sericin and agitated. Subsequently, 1 ml of sodium sulfide (10 mg/ml) was added until no further color change occurred in the mixture. The obtained solution was centrifuged at 6,000 rpm for 10 min, followed by 3 washes with deionized (DI) water and alcohol. The precipitate was collected and freeze-dried to obtain CuS-Se NPs.

To synthesize CuS-Se@TA-Fe NPs, 50 mg of CuS-Se NPs was mixed with 20 ml of DI water. Subsequently, 1 ml of TA solution (concentration of 40 mg/ml) was introduced and the mixture was stirred for 1 h. Following this, a solution of FeCl_3_·6H_2_O (1 ml, 5.3 mg/ml) was introduced while adjusting the pH to neutral. Finally, the precipitate was collected by centrifugation for 10 min.

The morphology of the CuS-Se@TA-Fe NPs were observed using transmission electron microscopy (TEM; JEM-2100, Tokyo, Japan). The composition and elemental distribution were measured using an x-ray energy-dispersive spectroscopy (EDS) detector. Chemical characterization was ascertained using x-ray photoelectron spectroscopy (XPS; Shimadzu DLD AXIS Ultra DLD, Japan). The samples were subjected to spectral analysis using an ultraviolet–visible (UV–vis) spectrophotometer (TU-1810, Shanghai, China), recording the UV–vis–NIR spectra. The Fourier transform infrared (FTIR) spectra of sericin, copper sulfide, CuS-Se, and CuS-Se@TA-Fe NPs were obtained utilizing an FTIR spectrometer (WQF-530, China).

### Fabrication and characterization of PCCuT

PVA (3 g) and CMCS (0.75 g) were solubilized in 30 ml of DI water at a temperature of 90 °C. After that, 300 mg of NPs was added and dispersed using ultrasound for 10 min. After that, 5 ml of 0.01 mM borax solution was added and mixed to obtain a composite hydrogel. The hydrogels were named PCCu or PCCuT, depending on whether CuS-Se NPs or CuS-Se@TA-Fe NPs were added, respectively. PC hydrogel was also prepared using the same method except without adding NPs. An EDS detector was employed to measure the composition and elemental distribution. Hydrogels were able to fuse into different shapes after being cut to examine the self-healing properties and shape plasticity.

The rheological properties of the hydrogel were evaluated using a rheometer (Anton Paar, Austria). The hydrogel was rapidly placed between the rheometer plates with a 1-mm gap, and its mechanical stability was assessed using a time sweep mode (10% strain, 1-Hz frequency).

Tensile tests were performed using a universal testing machine (Instron 5965, Massachusetts, USA), equipped with a 5-kN weight measuring element and a crosshead speed of 50 mm/min. All tests were conducted at room temperature.

To characterize the swelling behavior, the hydrogels were soaked in phosphate-buffered saline (PBS) (pH = 7.4) at 37 °C for 12 h. The swelling ratio was calculated using the following formula:

$$\text{Swelling ratio}\;\left(\%\right)=M_{1}/M_{0},$$ (1)

where *M*_1_ is the mass of the hydrogel after swelling and *M*_0_ is the initial mass of the hydrogel.

The preparation methods of PC, PCCu, and PCCuT hydrogels are described above. Equal amounts of hydrogel samples were immersed in simulated body fluid (SBF), ensuring that the samples were fully submerged. These samples were kept at 37 °C to simulate typical physiological conditions in the human body. At specific time points (1, 3, 5, and 7 d), hydrogel samples were extracted from the SBF solution. Excess liquid was carefully absorbed using absorbent paper, and the weight was measured. The calculation method for the residual mass at each time point is as follows:

$$\text{Weight remaining ratio}\;\left(\%\right)=\left(M_{0}-M_{n}\right)/M_{0}\times 100\%,$$ (2)

where *M*_0_ is the mass of the hydrogel at the beginning of the experiment and *M_n_* is the mass of the hydrogel at the corresponding time point.

The release of Cu^2+^ was quantified using an inductively coupled plasma optical emission spectrometer (ICP-OES; Agilent 5110, USA). A 1-ml hydrogel sample was immersed in PBS, and the supernatant was collected at predefined time intervals. The Cu^2+^ release profile at each time point was then analyzed using an ICP-OES.

### Evaluation of ROS scavenging ability in vitro

The free radical scavenging ability of CuS-Se NPs and CuS-Se@TA-Fe NPs was measured by employing DPPH and ABTS kits. In the presence of antioxidants, DPPH radicals were eliminated and the color (purple) of the reaction system became lighter and its absorbance at 515 nm decreased. PCCu and PCCuT hydrogels containing different concentrations of NPs were incorporated into the DPPH system. Absorbance was measured at 515 nm after 30 min of shielding from light.

$$\begin{array}{c} \text{DPPH scavenging ratio}\;\left(\%\right)=\\ \left[\left(A\;\text{blank}-A\;\text{sample}\right)/A\;\text{blank}\right]\times 100\%. \end{array}$$ (3)

The absorbance of the control and hydrogel groups is represented by *A* blank and *A* sample, respectively.

For the ABTS assay, when the test substance is added to the ABTS radical solution (blue-green color), its antioxidant capacity reacts with the ABTS radicals to discolor the solution, and the measured absorbance at 405 nm decreases. PCCu and PCCuT hydrogels containing different concentrations of NPs were incorporated into the ABTS system, and the absorbance at 405 nm was determined after 6 min of light protection at room temperature.

$$\begin{array}{c} \text{ABTS scavenging ratio}\;\left(\%\right)=\\ \left[\left(A\;\text{blank}-A\;\text{sample}\right)/A\;\text{blank}\right]\times 100\%. \end{array}$$ (4)

*A* blank and *A* sample represent the absorbance of the control and hydrogel groups, respectively.

### SOD-like activity assessment

The SOD activity of the hydrogels was evaluated by the nitrogen blue tetrazolium (NBT) colorimetric method. O_2_^•−^ was formed by the system of reactions between xanthine and xanthine oxidase, and in the presence of these superoxide anions, NBT was reduced to a blue-colored formazan, which strongly absorbed at 560 nm.

To determine the SOD activity of PCCu and PCCuT, hydrogels with different concentrations of NPs were reacted with a working solution containing NBT, and the UV–vis absorbance at 450 nm was recorded after incubation at room temperature for 30 min.

$$\text{Inhibition ratio}\;\left(\%\right)=\frac{\mathrm{Ap}-\mathrm{As}}{\mathrm{Ap}-\text{An}}\times 100\%,$$ (5)

where *Ap*, *An*, and *As* are the absorbance of the positive control, the negative control, and the sample, respectively.

### Oxygen generation experiment

To test the O_2_ generating capacity of the composite hydrogels, 1 mM hydrogen peroxide and PC, PCCu, and PCCuT hydrogels were mixed to 30 ml of PBS. O_2_ levels were measured every 30 s with a dissolved oxygen meter (JPBJ-608, REX, China).

### Measurement of photothermal capacity

NIR irradiation at 808 nm was utilized to assess the photothermal properties of the composite hydrogels PC, PCCu, and PCCuT. Each 1-ml hydrogel sample was subjected to NIR exposure at intensities of 0.4, 0.8, and 1.2 W/cm^2^ for 3 min. To determine the optimal laser intensity, we employed a thermal imaging camera (E8-XT, Wilsonville, USA) to capture the thermograms of the PC, PCCu, and PCCuT groups. The chosen optimal intensity was then used to assess the temperature decay of the hydrogel after repeated irradiation, enabling us to evaluate the material’s cyclic performance.

### In vitro antibacterial experiment

*Escherichia coli* and *Staphylococcus aureus* were inoculated into Luria–Bertani (LB) liquid medium to create a bacterial suspension with a concentration of 1.0 × 10^6^ CFU/ml. A volume of 100 μl of bacterial suspension (either *E. coli* or *S. aureus*) was evenly applied to a composite hydrogel (diameter = 10 mm and height = 5 mm), which was then exposed to NIR irradiation at an intensity of 0.8 W/cm^2^ and a wavelength of 808 nm for 3 min. Following this exposure, the bacterial suspensions were gathered and plated on solid LB medium, which was then incubated for 12 h at 37 °C. Photographs of the plates were taken, and the colonies were counted. In addition, bacterial suspensions were collected using the same procedure. Subsequently, live/dead fluorescent staining was applied to assess the antimicrobial efficacy of the composite hydrogel, and fluorescent microscopy (DMI8, Leica, Germany) was used to assess bacterial viability.

### Cytocompatibility

To obtain extracts, hydrogels from the different groups were macerated in complete cell medium for 48 h. HUVECs and RAW264.7 cells were incubated for 24 h in 96-well plates, using Dulbecco’s modified Eagle medium for both cell types. The collected extract solution then replaced the culture medium and was co-cultured for 1, 3, and 5 d. After incubation, Cell Counting Kit-8 (CCK-8) reagent was incorporated with the cells for 2 h. The absorption of the samples was determined at 450 nm with a microplate reader (Tecan Infinite M200 Pro, China). Additionally, the cytotoxicity of the hydrogel was evaluated through live/dead cell staining. Following the 1-, 3-, and 5-d culture periods, the cells were stained and visualized on a fluorescence microscope (DMI8, Leica, Germany).

### Hemolytic tests

To evaluate the hemocompatibility of PC, PCCu, and PCCuT hydrogels, 500 μl of rabbit blood with citrate added was centrifuged at 3,000 rpm for 10 min. After washing with PBS, the erythrocytes were gathered and diluted to attain a final concentration of 5% (v/v). Following this, the hydrogels of all groups were incorporated into 500 μl of cell suspension and co-cultured at 37 °C for 1 h. After centrifugation at 3,500 rpm for 5 min, the upper layer of the liquid was obtained, and the absorbance at 545 nm was determined using a microplate reader (Tecan Infinite M200 Pro, China). The positive control was 0.1% Triton X-100, while the negative control was PBS. The hemolysis ratio of the sample was calculated by the following formula:

$$\text{Hemolysis ratio}\;\left(\%\right)=\frac{\mathrm{Ah}-\mathrm{Ap}}{\mathrm{Aw}-\mathrm{Ap}}\times 100\%,$$ (6)

where *Ah*, *Ap*, and *Aw* represent the absorbance of the hydrogels, PBS, and 0.1% Triton X-100, respectively.

### In vitro scratch assay and tube formation assay

For the scratch assay, HUVECs were inoculated into 6-well plates at a density of 4 × 10^5^ cells per well. Once 100% cell fusion was achieved, a “scratch” without cells was made in the middle of the plate with a sterilized pipette tip. The extracts of PC, PCCu, and PCCuT hydrogels were then added to the corresponding wells, and photographs were then taken under an inverted-phase microscope to observe the changes in cells at 0, 24, and 48 h.

For tube formation assays, the matrix gel was first melted in a refrigerator at 4 °C for 5 h. Subsequently, 150 μl of the matrix gel was added to a 48-well plate and incubated for 30 min at 37 °C. After gelation, the cells were added to the well plates at a number of 3 × 10^4^ cells in each well. After a 6-h incubation, photographs of the cells were captured using a microscope. Statistical analysis of the mesh number, total tube length, and branch points was performed using ImageJ.

### Intracellular ROS scavenging assay

RAW264.7 cells were cultured with 3 different hydrogel extracts for 24 h. Subsequently, H_2_O_2_ was applied to each well at a concentration of 100 μM for a duration of 30 min. Then, it was carefully rinsed with PBS. Next, the cells were subjected to a 20-min incubation period with 2′,7′-dichlorodihydrofluorescein diacetate (DCFH-DA) staining solution. The ROS clearance status of the cells was subsequently photographed by fluorescence microscopy (DMI8, Leica, Germany).

### Macrophage polarization test

To investigate the macrophage polarization effects of the PCCuT hydrogel, immunofluorescence staining was performed. RAW264.7 cells were induced to differentiate into M1-type macrophages using lipopolysaccharide (1 μg/ml) and into M2-type macrophages with interleukin-4 (0.5 μg/ml) over 48 h. Afterward, the extracts from PC, PCCu, and PCCuT hydrogels were applied to the cells for an additional 48 h. M1-type macrophages were labeled with the biomarker inducible nitric oxide synthase (iNOS), while M2-type macrophages were labeled with CD206, incubated overnight at 4 °C. Confocal microscopy (IRX50, Sunny Optical Technology, China) was employed to capture images of the stained cells.

### In vivo hemostasis test

The Animal Ethics Committee of Southwest Medical University approved all animal experiments in this research. Hemostasis experiments were performed using 8-week-old Sprague Dawley rats weighing approximately 250 g. After anesthetizing the rats, 4.5 to 5.5 cm of the tail end was removed with surgical scissors to create a severed tail model. The same volume sizes of PC, PCCu, and PCCuT hydrogels were pressed onto the severed segments immediately after bleeding was observed; meanwhile, the control group was not subjected to any special treatment. Once hemostasis was achieved, the hemostasis time was recorded, and the bleeding mass was calculated by measuring the weight of the hydrogel and filter paper. Similarly, a 1.0-cm-long incision was made in the left hepatic lobe of rats to test the efficiency of hemostasis in the liver. After bleeding was observed, PC, PCCu, and PCCuT hydrogels of the same volume size were pressed into the wound. The hemostasis time was recorded after hemostasis was achieved, and the hemorrhage mass was calculated by weighing the hydrogels and the filter paper.

### In vivo diabetic infection wound-healing experiment

Animal models of type 2 diabetes were established using 8-week-old male Sprague Dawley rats. Briefly, the rats were fed a high-sugar and high-fat chow (Luzhou, Keyang Biotechnology Co., Ltd.) for 1 month, and then streptozotocin (30 mg/kg/d; Cayman Islands, USA) was injected intraperitoneally for 5 consecutive days. Blood glucose levels were monitored every 3 d using a glucometer (Accu-Chek Performa, Roche Diagnostics, USA). Feeding blood glucose ≥16.7 mM for 1 week was considered a model of type 2 diabetes.

Diabetic rats were divided into 6 groups, namely, the control, PC, PCCu, PCCuT, PCCu + NIR, and PCCuT + NIR groups. After anesthesia, 6 circular total skin wounds (diameter = 10 mm) were formed on the backs of the rats. Following that, the wounds were inoculated with 100 μl of *S. aureus* suspension at a concentration of 1 × 10^6^ CFU/ml; various hydrogels were placed into the infected wound sites, respectively. Wounds were captured on days 0, 3, 6, 9, 12, and 18. The wound size was counted with ImageJ, and the wound closure rate was calculated by utilizing the following formula:

$$\;\text{Wound closure rate}\;\left(\mathrm{WC}\%\right)=\frac{W_{0}-W_{t}}{W_{0}}\times 100\%,$$ (7)

where *W*_0_ represents the wound area on day 0 and *W_t_* represents the wound area on days 3, 6, 9, 12, and 18.

### Histological analysis

Tissue samples surrounding the wound were collected on days 6, 12, and 18 and fixed immediately in paraformaldehyde solution. Hematoxylin–eosin and Masson staining were performed to assess the impact of the PCCuT hydrogel on healing diabetic infected wounds. Additionally, immunofluorescence staining was conducted to analyze biomarkers, including IL-6, interleukin-10 (IL-10), CD68, CD86, CD206, CD31, alpha-smooth muscle actin (α-SMA), and vascular endothelial growth factor (VEGF).

### Statistical analysis

All results presented are mean ± standard deviation. One-way analysis of variance was employed for the experimental data, and asterisks indicate significant differences (*P < 0.05, **P < 0.01, ***P < 0.001, and ****P < 0.0001).

## Results and Discussion

### Characterization of CuS-Se@TA-Fe NPs

**Fig. 2.**
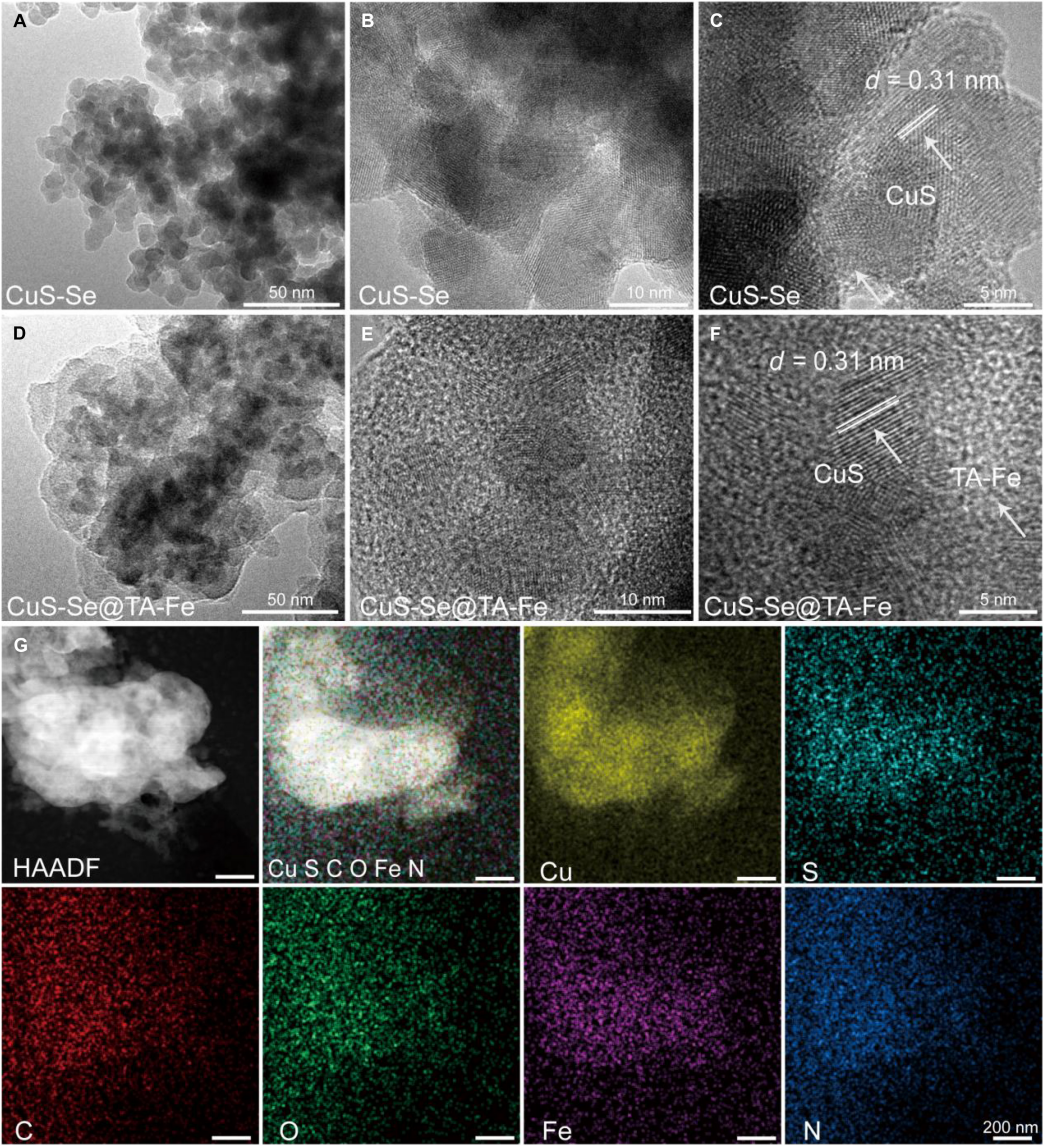
Morphological characteristics of CuS-Se NPs and CuS-Se@TA-Fe NPs. (A) Transmission electron microscopy (TEM) and (B and C) high-resolution Transmission electron microscopy (HR-TEM) images of CuS-Se NPs at different magnifications. (D) TEM and (E and F) HR-TEM images of CuS-Se@TA-Fe NPs at different magnifications. (G) High-angle annular dark-field scanning transmission electron microscopy (HAADF-STEM) of CuS-Se@TA-Fe NPs and elemental mapping of Cu, S, C, O, Fe, and N.

High-resolution TEM was applied to visualize the morphological appearance of CuS-Se NPs and CuS-Se@TA-Fe NPs. As shown in Fig. 2A and B, CuS-Se NPs with uniform diameters and near-spherical shapes are stacked together. High-resolution TEM showed that the lattice plane is about 0.31 nm, which corresponds to the (1 1 2) plane of CuS (Fig. 2C) [26]. Elemental mapping and EDS of CuS-Se NPs showed the elements of Cu, S, C, N, and O (Figs. S1 and S2). The morphological characteristics of CuS-Se@TA-Fe NPs are shown in Fig. 2D to F, where a thin coating can be found wrapped around the stacked CuS. In addition, the presence of an amorphous lattice is observed around the lattice of CuS, which clearly demonstrates the structural relationship between CuS-Se NPs and TA-Fe at the microscopic level. The high-angle annular dark-field scanning transmission electron microscopy of CuS-Se@TA-Fe NPs and the elemental mapping showed the uniform distribution of the Cu, S, C, O, Fe, and N elements (Fig. 2G). The presence of these elements was similarly verified by EDS in Fig. S3, confirming the preparation of CuS-Se@TA-Fe NPs.

**Fig. 3.**
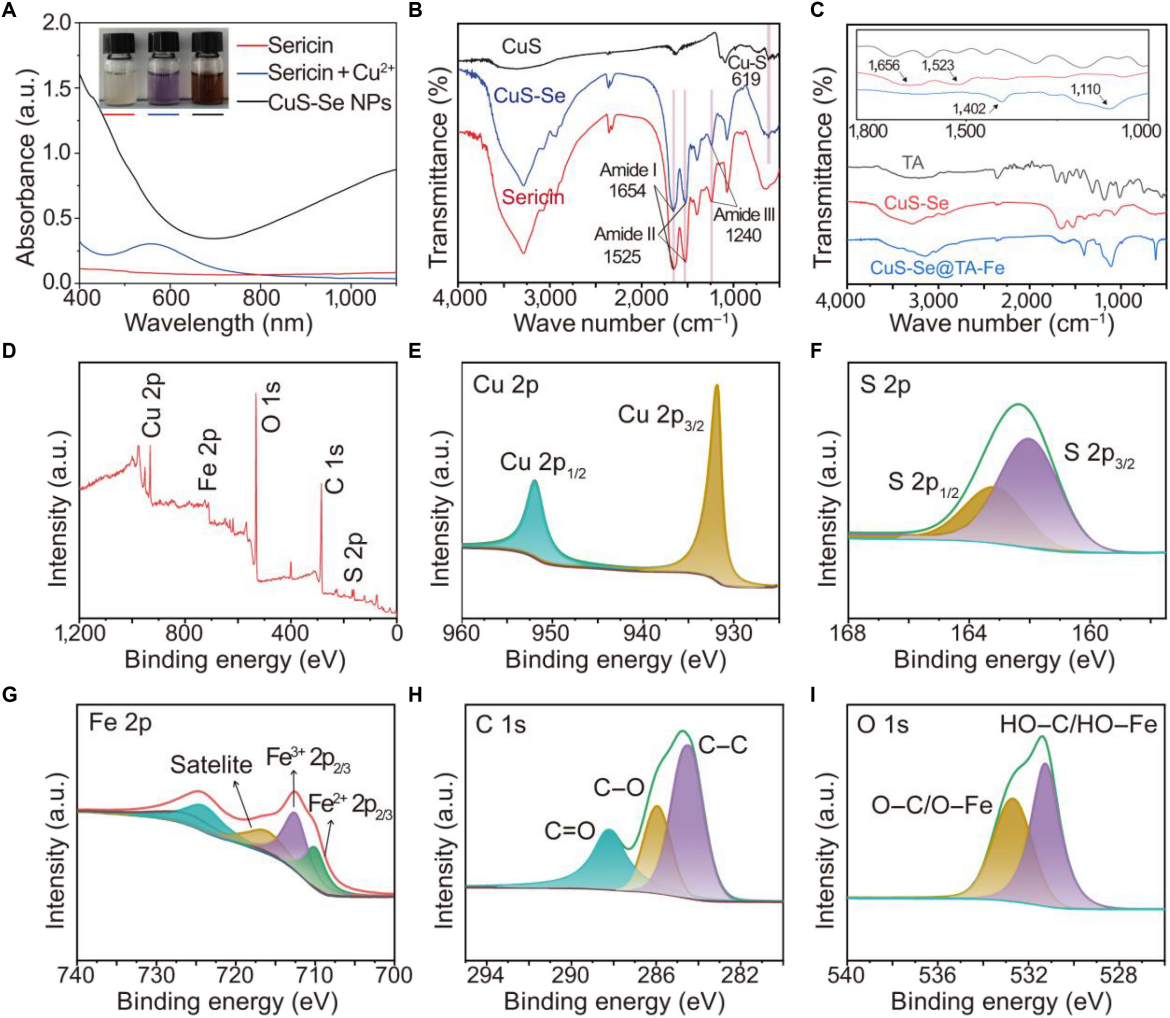
Characterization of CuS-Se@TA-Fe NPs. (A) Absorption spectra of sericin, sericin + Cu^2+^, and CuS-Se NPs in the ultraviolet–visible (UV–vis) range. (B) Fourier transform infrared (FTIR) spectra of sericin, CuS, and CuS-Se NPs. (C) FTIR spectra of tannic acid (TA), CuS-Se NPs, and CuS-Se@TA-Fe NPs. (D to I) X-ray photoelectron spectroscopy (XPS) measurement spectra and high-resolution XPS spectra of Cu 2p, S 2p, Fe 2p, C 1s, and O 1s signals.

To verify the elemental composition and valence characteristics of CuS-Se NPs and CuS-Se@TA-Fe NPs, we utilized the UV–vis, FTIR, and XPS techniques. The UV–vis spectrum of CuS-Se NPs showed pronounced absorption in the 600- to 1,100-nm range, confirming their NIR absorption capability (Fig. 3A) [27]. In the FTIR analysis, a peak at 619 cm^−1^ was identified as the stretching vibration of the Cu–S bond (Fig. 3B) [23]. The sericin spectrum displayed characteristic peaks at 1,654, 1,523, and 1,238 cm^−1^, corresponding to amides I, II, and III, respectively (Fig. 3B) [28]. The presence of both the Cu–S bond and amide peaks in the CuS-Se NP spectrum indicated successful synthesis. Notably, a remarkable shift in the stretching bands between 1,000 and 1,800 cm^−1^ was detected in the CuS-Se@TA-Fe NP spectra compared to the TA spectrum, suggesting interactions between the hydroxyl (–OH) groups and iron (Fe) ions (Fig. 3C).

Elemental composition and valence states in CuS-Se@TA-Fe NPs were examined using XPS. The survey spectra revealed the presence of Cu, S, Fe, C, and O (Fig. 3D). The binding energies of copper were recorded at 931.8 and 952.0 eV, which correspond to Cu 2p_3/2_ and Cu 2p_1/2_, respectively (Fig. 3E) [15]. The S 2p peaks at 162.0 and 163.2 eV are related to the Cu–S bond (Fig. 3F) [22], confirming the synthesis of CuS-Se NPs. For iron, the peaks at 710.1 and 712.6 eV indicated the presence of Fe(II) and Fe(III), respectively (Fig. 3G) [15]. The C 1s spectrum displayed 3 peaks, with 284.6 eV attributed to the C–C bond and the peaks at 286.0 and 288.23 eV referring to C–O and C=O bonds, respectively (Fig. 3H) [29]. The O 1s spectrum showed 2 peaks at 531.7 and 532.7 eV, linked to O–C/O–Fe and HO–C/HO–Fe, respectively (Fig. 3I) [30]. Collectively, these experiments confirmed the synthesis of CuS-Se@TA-Fe NPs.

### Characterization of the hydrogels

**Fig. 4.**
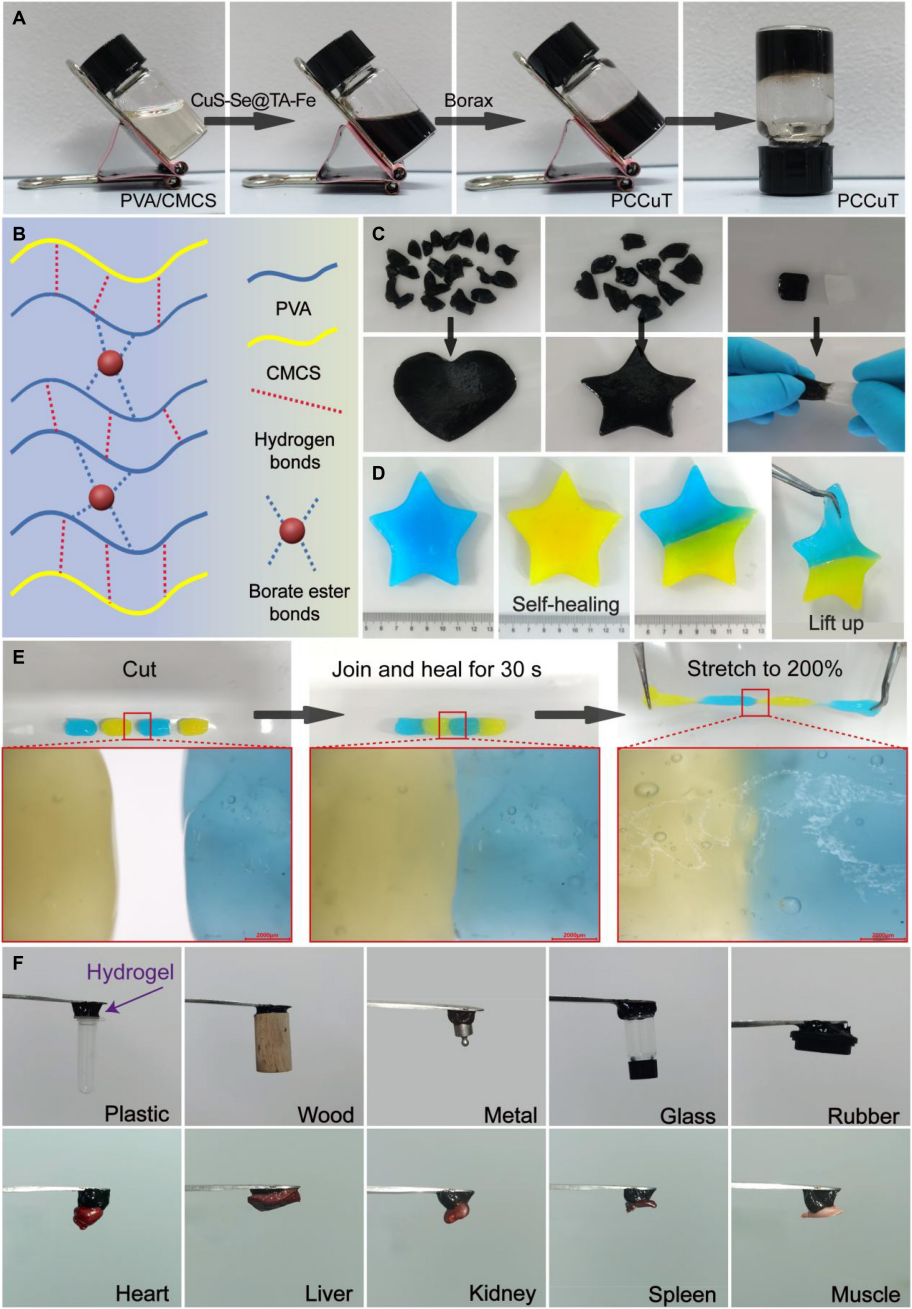
Self-healing and adhesion and properties of PCCuT. (A) Rapid cross-linking process of the PCCuT hydrogel. (B) Schematic diagram of the hydrogel cross-linking network. (C) Morphological plasticity of the PCCuT hydrogel. (D) Self-healing properties of hydrogels. (E) Stretching of hydrogels after self-healing and characterization of the interface under a microscope. (F) Adhesion of the PCCuT hydrogel for biological and non-biological tissues.

As shown in Fig. 4A, the formation of hydrogels was challenging due to the weak hydrogen bonding between PVA and CMCS. However, the addition of borax facilitated rapid hydrogel formation. This is due to the cross-linking of the hydrogen bonds possessed by PVA and CMCS with the borate ester bonds contained in borax, forming a dual network hydrogel composed of PVA and CMCS. The EDS elemental mapping shows that the elements in the PCCuT hydrogel are evenly distributed throughout the hydrogel network, with no significant aggregation observed (Fig. S4). Since the borate ester bonds can undergo dynamic bonding and deconjugation at room temperature, the hydrogel has an excellent self-healing property and shape plasticity (Fig. 4B) [31]. Chopped hydrogels were placed in molds, and complete pentagonal and heart-shaped hydrogels were rapidly formed to investigate the self-healing behavior of the PCCuT hydrogel. No breakage occurred when stretching the healed hydrogel, demonstrating its good shape plasticity (Fig. 4C).

In order to observe the self-healing properties of the hydrogels more intuitively, 2 pieces of stained pentagonal hydrogels were cut so that they could be quickly spliced together (Fig. 4D). The experiments described above likewise demonstrated the shape plasticity of hydrogels, which have important potential for coping with irregular wounds. In addition, to further demonstrate the self-healing ability of the hydrogels, 4 segments of the stained hydrogels were put together and the morphology of the joining surfaces was monitored by microscope (Fig. 4E). After 30 s, the length of the hydrogel became twice as long as the original and still did not break. The connecting line between the yellow and blue hydrogels was blurred under the microscope, demonstrating its self-healing property from a new angle. Due to the presence of a large number of hydrogen bonds, PCCuT hydrogels have excellent adhesion properties to common abiotic tissues, including plastics, wood, metals, glass, and rubber. Additionally, adhesion tests were further performed using rat heart, liver, kidney, spleen, and muscle, and the PCCuT hydrogel similarly showed good adhesion properties to biological tissues (Fig. 4F). Furthermore, the rheological experiments showed that the PCCuT hydrogel’s *G*′ (storage modulus) was higher than *G*″ (loss modulus), indicating the mechanical stability of the hydrogel (Fig. S5).

As shown in Fig. S6, we conducted a tensile test to evaluate the mechanical strength of the hydrogel. With the incorporation of NPs, the tensile strength of the PCCuT hydrogel was enhanced (0.46 MPa), accompanied by a higher Young’s modulus (4,667 kPa). This improvement may be attributed to the strengthening of the hydrogel network’s cross-linking due to the presence of TA [32].

To evaluate the stability of the hydrogel cross-linked network, swelling and degradation experiments were performed. The composite hydrogels were soaked in PBS buffer at 37 °C to assess their swelling ratio (Fig. S7). After the addition of NPs, the maximum swelling ratio of the PCCuT hydrogel slightly decreased. This reduction is attributed to the increase in solid content, and the presence of TA led to tighter cross-linking, leaving less space for water storage. In addition, to evaluate the degradation ability of the PCCuT hydrogel, it was immersed in SBF to assess the hydrogel’s stability. As illustrated in Fig. S8, the remaining mass of the hydrogel progressively decreased over time, reaching only 20.6% at day 7, thereby demonstrating its excellent degradation performance.

### In vitro free radical scavenging capacity

**Fig. 5.**
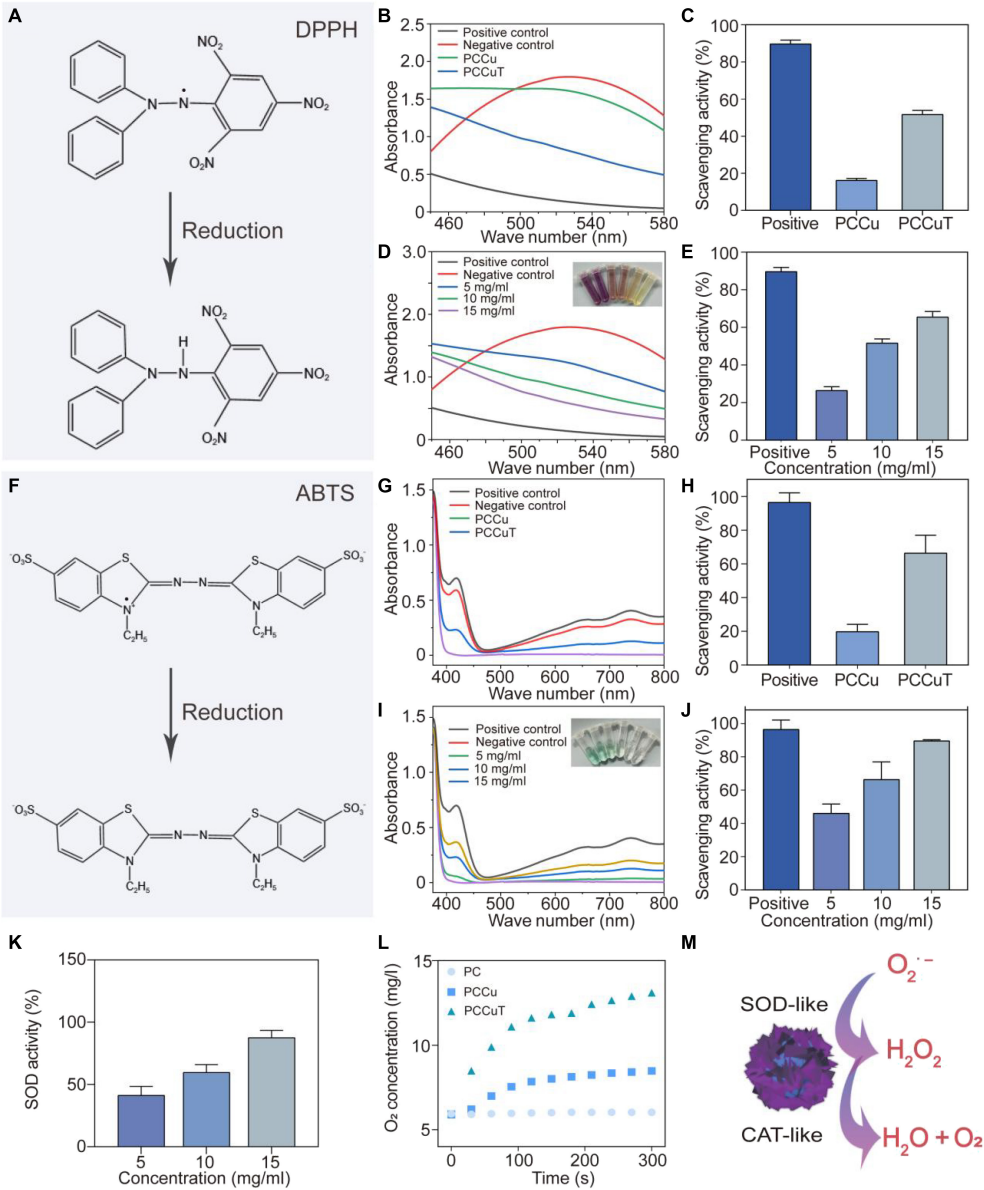
Free radical scavenging capacity and nanoenzymatic effect of the PCCuT hydrogel. (A) Redox mechanism of free radical scavenging by 1,1-diphenyl-2-pyridylhydrazine (DPPH). (B and C) DPPH determination of the UV absorption curves and scavenging rates of different kinds of hydrogels. (D and E) DPPH determination of the UV absorption curves and scavenging rates of PCCuT hydrogels at different CuS-Se@TA-Fe NP concentrations. (F) Redox mechanism of 2,2′-azobis(3-ethylbenzothiazoline-6-sulfonic acid) (ABTS) radical scavenging. (G and H) ABTS determination of the UV absorption curves and scavenging rates of different types of hydrogels. (I and J) ABTS determination of the UV absorption curves and scavenging rates of PCCuT hydrogels at different CuS-Se@TA-Fe NP concentrations. (K) SOD activity determination of different hydrogels. (L) O_2_ generation activity assay of different hydrogels. (M) Schematic representation of the SOD-like and CAT-like activities of CuS-Se@TA-Fe NPs.

During the healing process of diabetic wounds, a large number of free radicals are produced, and these excess free radicals promote an inflammatory response, damage DNA, attack cell membranes, and are detrimental to wound recovery and healing [33]. Therefore, the removal of excess free radicals is an important step in diabetic wound healing. This study tested the ability of the PCCuT hydrogel to scavenge free radicals by DPPH free radical scavenging assay. The redox mechanism of the DPPH radical scavenging assay is shown in Fig. 5A. In the existence of antioxidants, DPPH radicals were eliminated, the color of the solution (purple) became lighter, and its absorbance at 515 nm decreased. As shown in Fig. 5B, the PCCuT hydrogel had a lower absorption peak at 515 nm compared to the PCCu hydrogel, indicating better scavenging of DPPH radicals. The free radical removal capacity of the PCCuT hydrogel was obviously higher than that of the PCCu hydrogel due to the addition of the TA-Fe MPN (Fig. 5C). Furthermore, as the concentration of NPs in the PCCuT hydrogel increased, the absorption peak at 515 nm of the mixed solution diminished, and the DPPH radical scavenging rate gradually increased (Fig. 5D and E).

Similarly, the ABTS free radical scavenging assay is another classic method for assessing free radical scavenging capacity. Figure 5F shows the redox mechanism of the ABTS radical scavenging experiment. After the PCCuT hydrogel came into contact with the ABTS free radical solution, the antioxidant effect of the hydrogel changed the color of the reaction system from blue-green to light, resulting in a smaller UV absorption peak at 405 nm (Fig. 5G). Furthermore, the scavenging of ABTS radicals was better with increasing concentration of CuS-Se@TA-Fe NPs in PCCuT hydrogels (Fig. 5I to J). The above experiments showed that PCCuT hydrogel has a significant effect in scavenging free radicals, which is important for diabetic wound healing.

### SOD-like activity and oxygen generation detection

The SOD-like nanoenzymatic activity can promote the conversion of O_2_^•−^ to H_2_O_2_. In contrast, the CAT-like nanoenzymatic activity can decompose H_2_O_2_ to generate H_2_O and O_2_ to alleviate hypoxia in diabetic wounds due to impaired local angiogenesis. At the same time, SOD-like and CAT-like nanoenzymatic activities can remove excessive ROS, thereby reducing local inflammation and promoting diabetic wound healing. Here, the NBT colorimetric assay is used to detect the SOD-like nanoenzyme activity of PCCu and PCCuT hydrogels. In the presence of superoxide anions, NBT is reduced to formazan, which has a strong absorption at 560 nm. As shown in Fig. 5K, both PCCu and PCCuT hydrogels with SOD-like nanoenzymatic activity reduced the superoxide anion, which decreased the absorption at 560 nm. In addition, the SOD-like nanoenzymatic activity of PCCu and PCCuT hydrogels was positively correlated with the content of CuS NPs.

In order to detect the CAT-like nanoenzymatic activity of the PCCuT hydrogel, the composite hydrogel was mixed with H_2_O_2_, and the change in oxygen content was detected. As shown in Fig. 5L, the oxygen content of the PCCuT hydrogel group at 300 s was 13.1 mg/l, which was higher than those of the PCCu hydrogel and PC hydrogel groups. This is mainly due to the catalytic properties of the TA-Fe coating. The above results indicate that CuS-Se@TA-Fe NPs have SOD-like and CAT-like nanoenzyme activities, which can scavenge ROS and provide oxygen to wound tissue (Fig. 5M).

### Photothermal properties of hydrogels

To investigate the photothermal properties of the composite hydrogels, we exposed them to NIR lasers and monitored temperature changes using infrared thermography (Fig. S9A). As illustrated in Fig. S9B, the temperature of the PC hydrogel remained relatively constant under varying NIR powers (0.4, 0.8, and 1.2 W/cm^2^) for 3 min, while PCCu and PCCuT hydrogels exhibited significant temperature increases. The platform temperature of PCCu and PCCuT hydrogels increases with the rise in NIR power (Fig. S9C to E). The introduction of TA-Fe notably enhanced the photothermal conversion efficiency of the PCCuT hydrogel compared to that of the PCCu hydrogel. Under 0.8 W/cm^2^ irradiation, the PCCuT hydrogel reached 54.3 °C in 2 min and 56.9 °C in 3 min. Furthermore, NIR “switching” tests were also carried out to determine the photothermal stability of the PCCuT hydrogel (Fig. S9F). After 5 cycles of irradiation followed by spontaneous cooling, the maximum temperature remained relatively stable, indicating consistent photothermal conversion performance. These findings demonstrate that the PCCuT hydrogel possesses high photothermal conversion efficiency, supporting its potential for antimicrobial therapy in diabetic wounds.

### In vitro antimicrobial properties

**Fig. 6.**
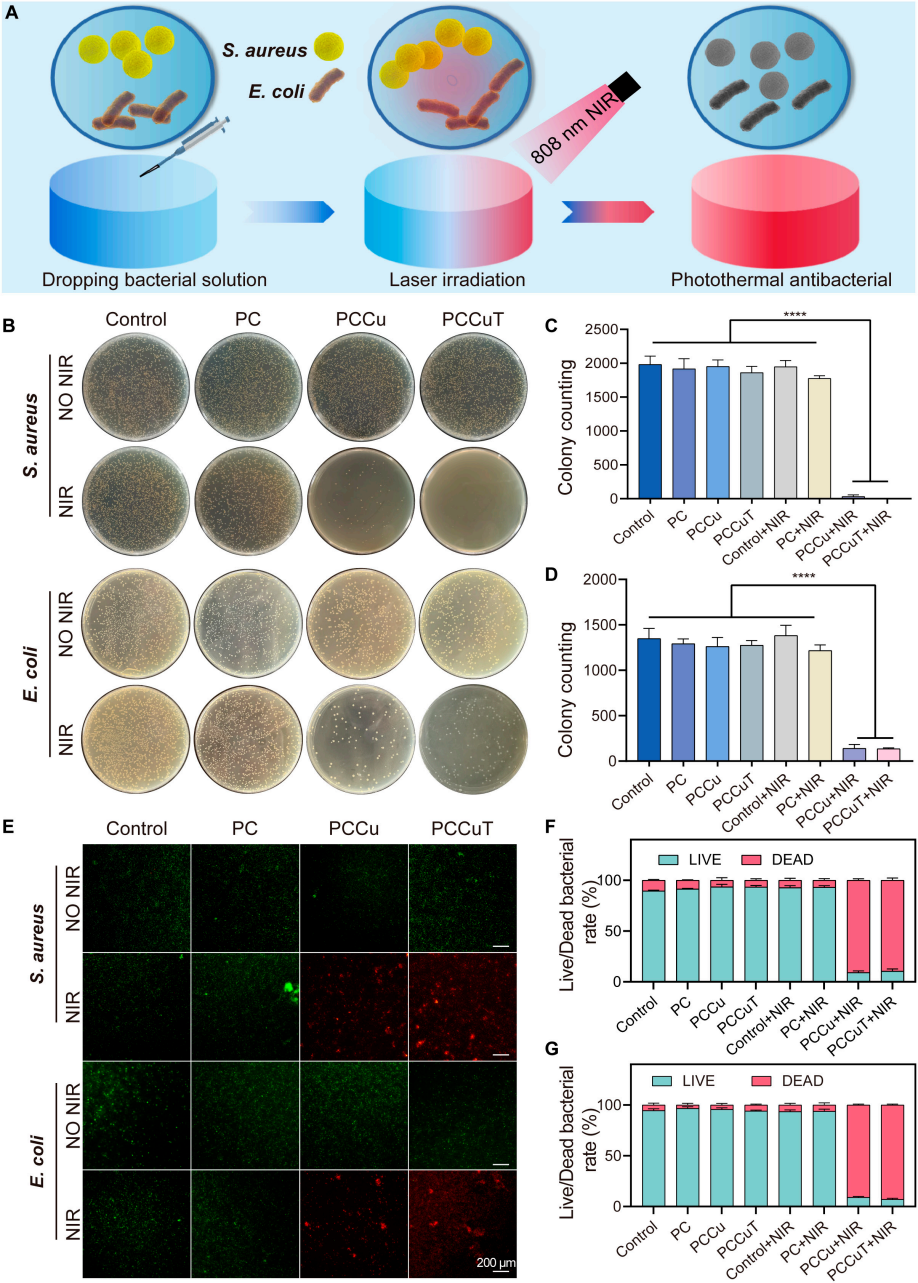
In vitro antimicrobial properties of PC, PCCu, and PCCuT hydrogels. (A) Schematic representation of the hydrogel’s photothermal antibacterial ability. (B) Images of colony formation after near-infrared (NIR; 808 nm, 0.8 W/cm^2^) irradiation of PC, PCCu, and PCCuT hydrogels. (C) Statistics of *Staphylococcus aureus* colony counts. (D) Statistics of *Escherichia coli* colony counts. (E) Live/dead stained images of *S. aureus* and *E. coli*. Live/dead bacterial rate of (F) *S. aureus* and (G) *E. coli*.

The antimicrobial properties of wound dressings are essential for the healing of infected wounds. The photothermal antimicrobial capability of the hydrogels was assessed using the diffusion plate method and bacterial live/dead staining. As illustrated in Fig. 6A, the bactericidal mechanism of the PCCuT hydrogel involves high temperatures, causing permanent denaturation of bacterial proteins. *E. coli* and *S. aureus* were used as model organisms for antimicrobial tests. Notably, the colonies of both bacteria in the PCCu and PCCuT groups were significantly reduced when exposed to NIR light at 0.8 W/cm^2^ (Fig. 6B to D), demonstrating a strong bactericidal effect.

To further confirm the antimicrobial properties, we performed live/dead bacterial staining after NIR irradiation. The bacterial suspensions collected showed that the control, PC, PCCu, and PCCuT groups displayed prominent green fluorescence (indicating live bacteria). In contrast, the PCCu + NIR and PCCuT + NIR groups primarily exhibited red fluorescence (indicating dead bacteria), as shown in Fig. 6E to G. These findings support the conclusion that PCCu and PCCuT hydrogels possess substantial photothermal antimicrobial capabilities, consistent with the plate count results. Overall, the PCCuT hydrogel demonstrates excellent photothermal conversion efficiency and antimicrobial effectiveness, indicating its potential for treating diabetic infected wounds.

### In vitro cytocompatibility and hemocompatibility investigations

Excellent biocompatibility and hemocompatibility are essential and necessary properties of an ideal wound dressing. In our study, the cytocompatibility of the hydrogel was assessed by CCK-8 analysis and live/dead cell staining. The hydrogel extracts were incubated with HUVECs and RAW264.7 cells. Similar to the cells in the control group, the cells in all hydrogel-treated groups exhibited comparable growth densities, suggesting no specific effect on the growth of HUVECs (Fig. S10A). In addition, there were no significant differences in RAW264.7 cells cultured for 1, 3, and 5 d in the hydrogel-treated groups compared to the control (Fig. S10C). To further assess the biocompatibility of the hydrogels, we conducted the CCK-8 assay to evaluate cell viability. After 1, 3, and 5 d of co-culture with HUVECs and RAW264.7 cells, there was no significant variation in cell survival between the hydrogel-treated group and the control group (Fig. S10B and D), indicating excellent cytocompatibility. The ICP-OES results indicated that the release of Cu^2+^ increased gradually over 48 h, without any noticeable burst release. At 48 h, the released copper ion concentration was 14.8% (1.8 μg/ml), which remains below the threshold for potential toxicity (Fig. S11) [34].

Moreover, the blood compatibility of the composite hydrogel was further researched. When exposed to less compatible substances, erythrocytes rupture, leading to hemolysis [20]. Here, erythrocytes were incubated with PC, PCCu, PCCuT, PBS, and 0.1% Triton X-100. The PBS group stands for the negative control group, and the Triton X-100 group stands for the positive control group. The results showed that each group of hydrogel-treated erythrocytes maintained good morphology with a typical biconcave disk shape (Fig. S10E). The hemolysis ratios of the hydrogel-treated groups were all below the international standard of 5%, indicating good hemocompatibility of the PC, PCCu, and PCCuT hydrogels (Fig. S10F).

### Migration assay and tube formation assay

Wounds in diabetic patients usually suffer from vascular lesions due to persistently elevated blood glucose levels and a local inflammatory environment, resulting in inadequate blood supply to the wound site and impeding wound healing [35]. Therefore, an effective diabetic wound dressing should promote angiogenesis at the wound site. Copper is a vital element in human growth and development. A large number of research studies have demonstrated that copper assumes a crucial function in enhancing the expression and secretion of VEGF and related cytokines, which ultimately leads to the promotion of angiogenesis [36]. The cell scratch experiment revealed that the migrated area of HUVECs in the PCCu and PCCuT groups was significantly larger than that in the control and PC groups (Fig. S12A and B). This result suggests that the PCCu and PCCuT groups have the ability to promote the migration of HUVECs.

The tube-forming assay aimed to simulate the ability of HUVECs to remodel the extracellular matrix using Matrigel [37]. Figure S12C demonstrates that both the PCCu group and the PCCuT group formed more tubelike structures. Moreover, the PCCu and PCCuT groups displayed higher numbers of nodes and meshes and overall length of tube formation compared to the control and PC groups (Fig. S12D to F). The above experimental results indicate that the PCCuT hydrogel has a significant impact on angiogenesis, thereby demonstrating its potential to be an ideal diabetic wound dressing.

### ROS clearance and macrophage immunoregulatory properties

DCFH-DA was applied for the detection of intracellular ROS by adding H_2_O_2_ to the culture medium to simulate the environment of the cells after stimulation. As shown in Fig. S13, the fluorescence intensity was significantly increased in the positive control group compared to that in the control group, indicating that intracellular ROS were significantly elevated. The fluorescence intensities of the PC and PCCu groups did not differ substantially from those of the positive control group. However, the fluorescence intensity of the PCCuT group was significantly lower, indicating that the PCCuT hydrogel can remove intracellular ROS.

Macrophages are multifunctional immune cells that play a crucial role in regulating tissue homeostasis, defending against pathogens, and promoting wound healing [38]. Influenced by the immune microenvironment, macrophages can differentiate into M1-type and M2-type macrophages [39]. During wound healing, M1-type macrophages secrete pro-inflammatory cytokines, such as tumor necrosis factor alpha, and produce high levels of iNOS [4]. M2-type macrophages secrete cytokines with anti-inflammatory properties, such as IL-10, and exhibit a high expression of CD206 [40]. However, the microenvironment of wounds in diabetic patients is altered, preventing the transition of macrophages from pro-inflammatory M1-type macrophages to anti-inflammatory M2-type macrophages [41], which is detrimental to wound healing. Here, to verify the immunomodulatory effect of the PCCuT hydrogel on macrophage phenotypes, immunofluorescence staining was used to assess the effects of the PCCuT hydrogel on macrophage M1/M2 polarization.

Macrophages were induced into M1 and M2 types of macrophages by lipopolysaccharide and interleukin-4, with iNOS and CD206 selected to label them, respectively. As shown in Fig. S14, the PCCuT group demonstrated substantially elevated levels of CD206 fluorescence intensity compared to the negative control, PC, and PCCu groups. Conversely, the fluorescence intensity of iNOS in the PCCuT group was significantly lower than that in the negative control, PC, and PCCu groups (Fig. S15). The above results indicate that the PCCuT hydrogel has the ability to scavenge intracellular ROS and activate macrophage differentiation into the M2 type, which is essential for inhibiting the inflammatory response and promoting wound healing in diabetic wounds.

### Evaluation of hemostatic efficiency in vivo

Hemostasis is vital in the initial stages of wound healing. In our study, we evaluated the hemostatic properties of PC, PCCu, and PCCuT hydrogels using a rat tail-break model and a liver injury model (Fig. S16A). As depicted in Fig. S16B, images captured at various time points after hydrogel application revealed that both hemostasis time and bleeding volume were considerably reduced in the PC and PCCu groups compared to those in the control group, highlighting the hemostatic effect of CMCS in the hydrogel. The PCCuT group exhibited significantly less bleeding and shorter hemostasis times (Fig. S16C and D). In the rat liver hemostasis model, both the PC and PCCu groups demonstrated effective hemostatic capabilities; however, the PCCuT group achieved the shortest hemostasis time (65 s) and the lowest bleeding volume (104 mg) (Fig. S16F and G). The PCCuT hydrogel exhibits a higher hemostatic effect, primarily due to the following aspects: (a) Its excellent adhesiveness allows the hydrogel to firmly adhere to the wound site, forming a stable interface that effectively prevents blood loss. (b) The internal porous structure increases the surface area, promotes blood absorption, and helps remove excess fluid around the wound, thereby accelerating blood clotting. (c) The Fe^3+^ released from the PCCuT hydrogel promote the aggregation and adhesion of blood cells, enhancing the coagulation process [42]. (4) The polyphenolic groups in TA interact with blood components, further accelerating the coagulation process [43]. Through these integrated mechanisms, the PCCuT hydrogel demonstrates a stronger hemostatic effect compared to PC and PCCu.

### Diabetic wound-healing assay

**Fig. 7.**
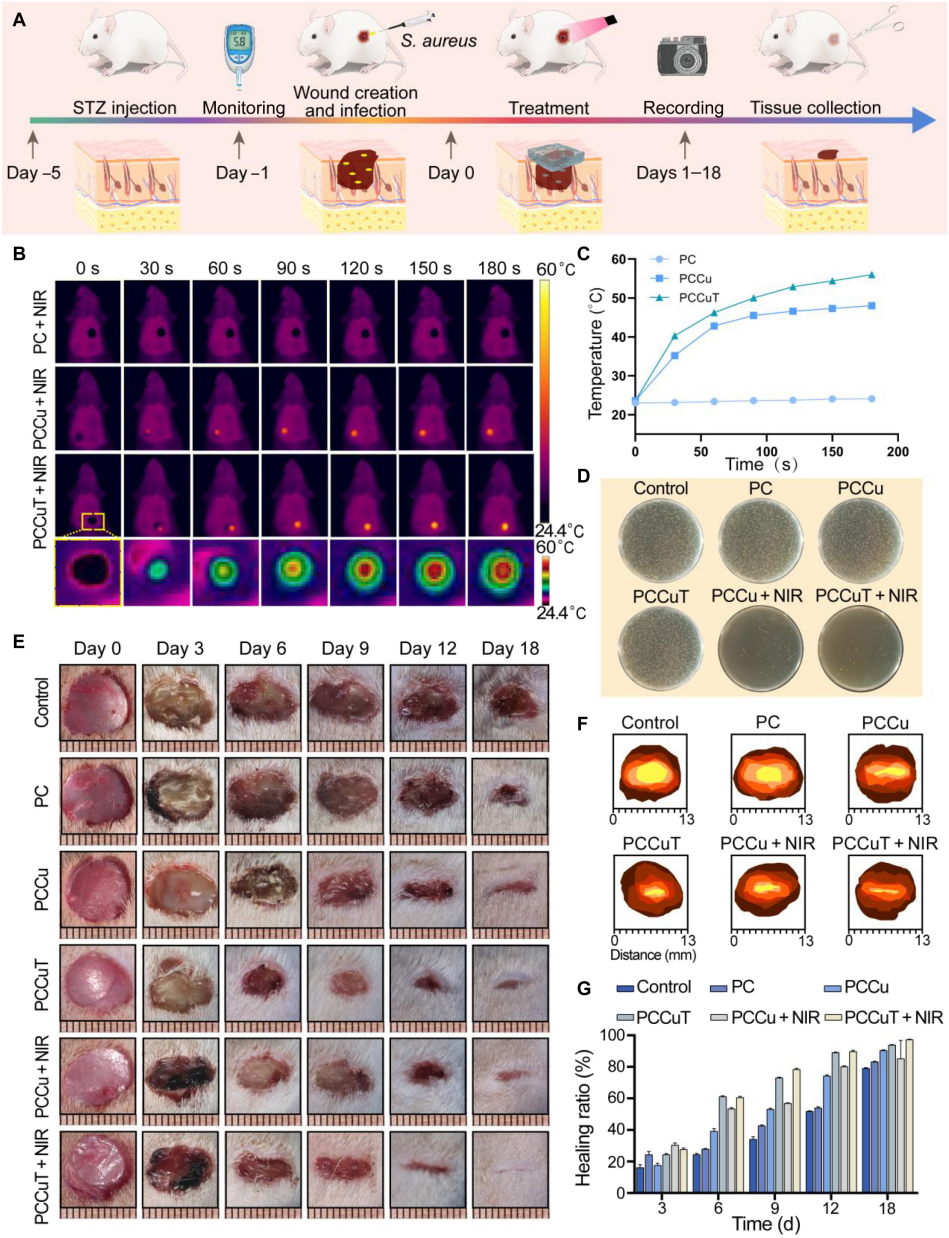
Ability of the PCCuT hydrogel to promote wound healing in vivo. (A) Schematic diagram of the established diabetic infected wounds and the hydrogel treatment. (B) Infrared images and (C) photothermometry curves of the PC group, PCCu group, and PCCuT group under NIR irradiation. (D) Images of colonies formed by fluid collected from the wound. (E) Representative images of wounds in diabetic rats on days 0, 3, 6, 9, 12, and 18. (F) Diagrammatic representation of the process of wound healing. (G) Quantitative analysis of the wound-healing rate in each group on different days. STZ, streptozotocin.

In vitro experiments demonstrated the excellent photothermal antimicrobial, inflammation-modulating, ROS scavenging, and angiogenic capabilities of the PCCuT hydrogel. The potential of the PCCuT hydrogel for repairing diabetic infected wounds was further investigated using a model of full-thickness skin defects in type 2 diabetic rats (Fig. 7A). The successful establishment of the diabetic model was demonstrated by testing the blood glucose levels of diabetic rats (Fig. S17). During the photothermal treatment of infected wounds on the backs of diabetic rats, thermography was employed to record temperature changes (Fig. 7B). The PCCuT hydrogel increased from 23.6 to 52.9 °C within 2 min after NIR irradiation (Fig. 7C). The bacterial fluid from the wound was then collected and cultured on LB solid medium. There was a lower number of colonies in the PCCu + NIR group and PCCuT + NIR group, suggesting effective photothermal antimicrobial activity (Fig. 7D). Then, the therapeutic efficacy of the PCCuT hydrogel was further verified by recorded the wound-healing progress over 18 d. As shown in Fig. 7E, pronounced pus could be observed in the control, PC, PCCu, and PCCuT groups on day 3. However, no pus was observed in the PCCu + NIR and PCCuT + NIR groups. On the sixth day, the wound recovery in the PCCuT, PCCu + NIR, and PCCuT + NIR groups was significantly better than that in the control, PC, and PCCu groups, which may be closely related to the anti-inflammatory, antioxidant ability, and photothermal antimicrobial effect of the hydrogel. The PCCu + NIR group still demonstrated a significant wound-healing effect, which may be attributed to the copper ions released, promoting angiogenesis. At the same time, no pronounced pus formation was observed at the wound site, indicating effective bacterial infection clearance. On the 18th day, the wounds in the PCCuT + NIR group were essentially healed, whereas visible wounds were observed in the remaining groups. In addition, a topographical map of wound healing was drawn to better visualize the changes in wound area on different days (Fig. 7F). The wound area in each group was quantitatively analyzed (Fig. 7G), which again showed the best wound-healing rate in the PCCuT + NIR group. The findings suggest that the PCCuT hydrogel possesses remarkable characteristics for promoting the recovery of diabetic infected wounds.

### Histopathological analysis

**Fig. 8.**
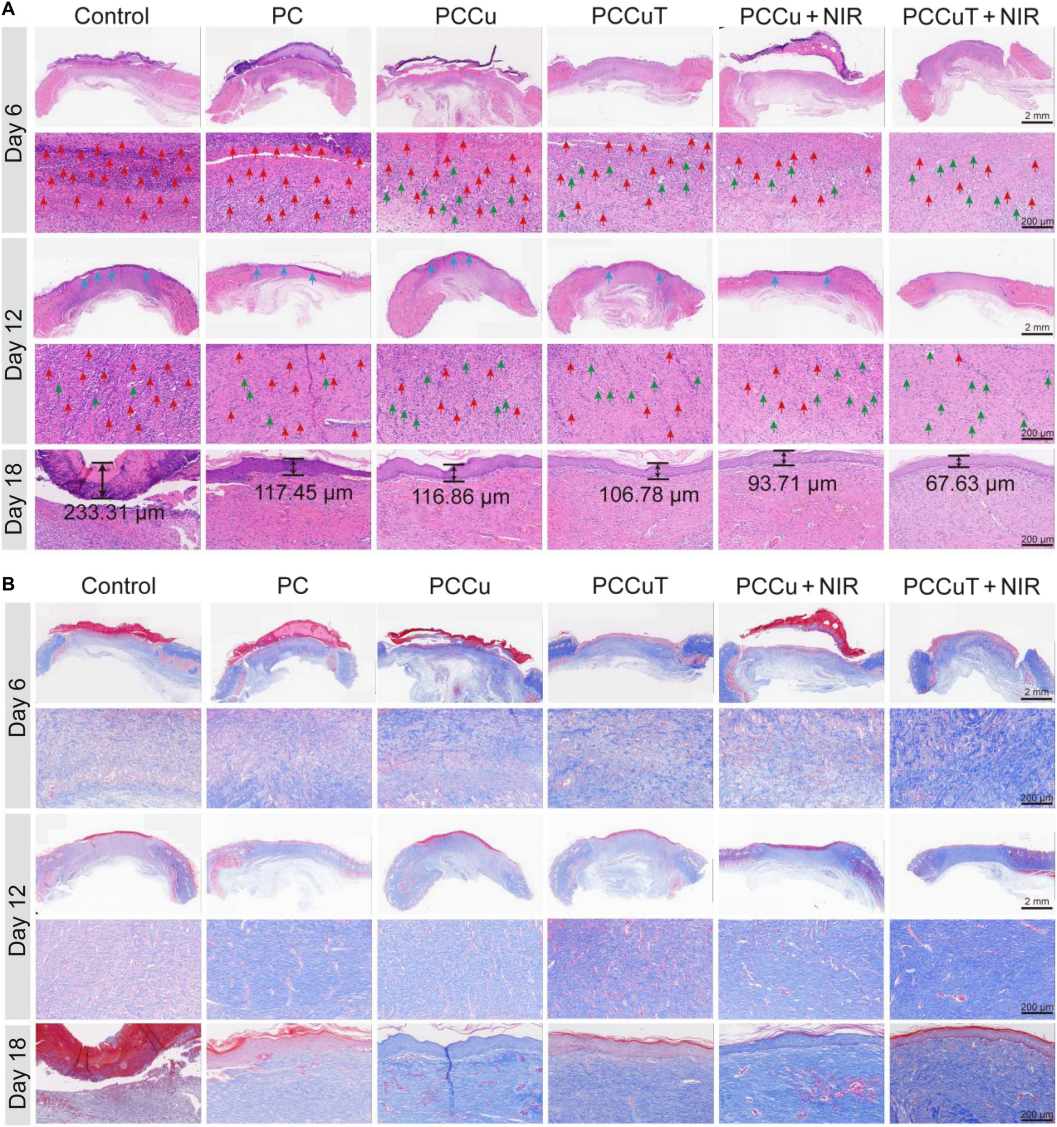
In vivo histopathologic analysis. (A) Hematoxylin–eosin (H&E) and (B) Masson staining of the wound areas on days 6, 12, and 18. Red arrows indicate inflammatory cells, green arrows indicate neoplastic capillaries, blue arrows indicate neoplastic epithelium where it is not smooth, and black arrows indicate epithelial borders.

To further investigate the therapeutic effects of the PCCuT hydrogel on skin tissue regeneration, skin tissues from diabetic rats were collected on days 6, 12, and 18. The tissues were then stained with hematoxylin–eosin and Masson’s trichrome. On day 6, there were more inflammatory cells in the control group and PC group. In contrast, the PCCuT + NIR group had the fewest inflammatory cells (Fig. 8A). Compared with the control group and PC group, new capillaries appeared in the PCCu, PCCuT, PCCu + NIR, and PCCuT + NIR groups, which is related to the procapillary angiogenesis effect of copper ions. On day 12, the degree of inflammation in the PCCuT + NIR group was significantly lower than those of other groups, and the number of new capillaries was higher. The new epithelium in the PCCuT + NIR group was smoother, while other groups had epithelial irregularities, indicating better wound healing. In the process of wound healing, the thickness of epithelial tissue undergoes a transition from thin to thick and subsequently gradually thins again during the later stages [23]. On day 18, the thickness of the epithelium in the PCCuT + NIR group was 67.63 μm, which was thinner than those of the control (233.31 μm), PC (117.45 μm), PCCu (116.86 μm), PCCuT (106.78 μm), and PCCu + NIR (93.71 μm) groups. The above results indicated that the wound-healing effect was better in the PCCuT + NIR group.

Collagen plays a crucial role in the wound-healing process as it is the main component of skin tissue [44]. It enhances the elasticity and toughness of wounds, thereby promoting wound healing and scar repair. To assess the amount of epithelial collagen deposition in diabetic wounds and surrounding tissues, Masson staining was performed (Fig. 8B). Notably, on days 6, 12, and 18, the PCCuT + NIR group exhibited the darkest staining compared to the other groups, indicating its superior healing effect. These findings demonstrate that the PCCuT + NIR group has the most effective results in healing wound tissue. This is mainly due to the following: (a) the photothermal effect kills wound bacteria, (b) CuS-Se@TA-Fe NPs remove excess ROS, and (c) copper ions promote capillary angiogenesis.

### In vivo anti-inflammatory and angiogenic capacity

**Fig. 9.**
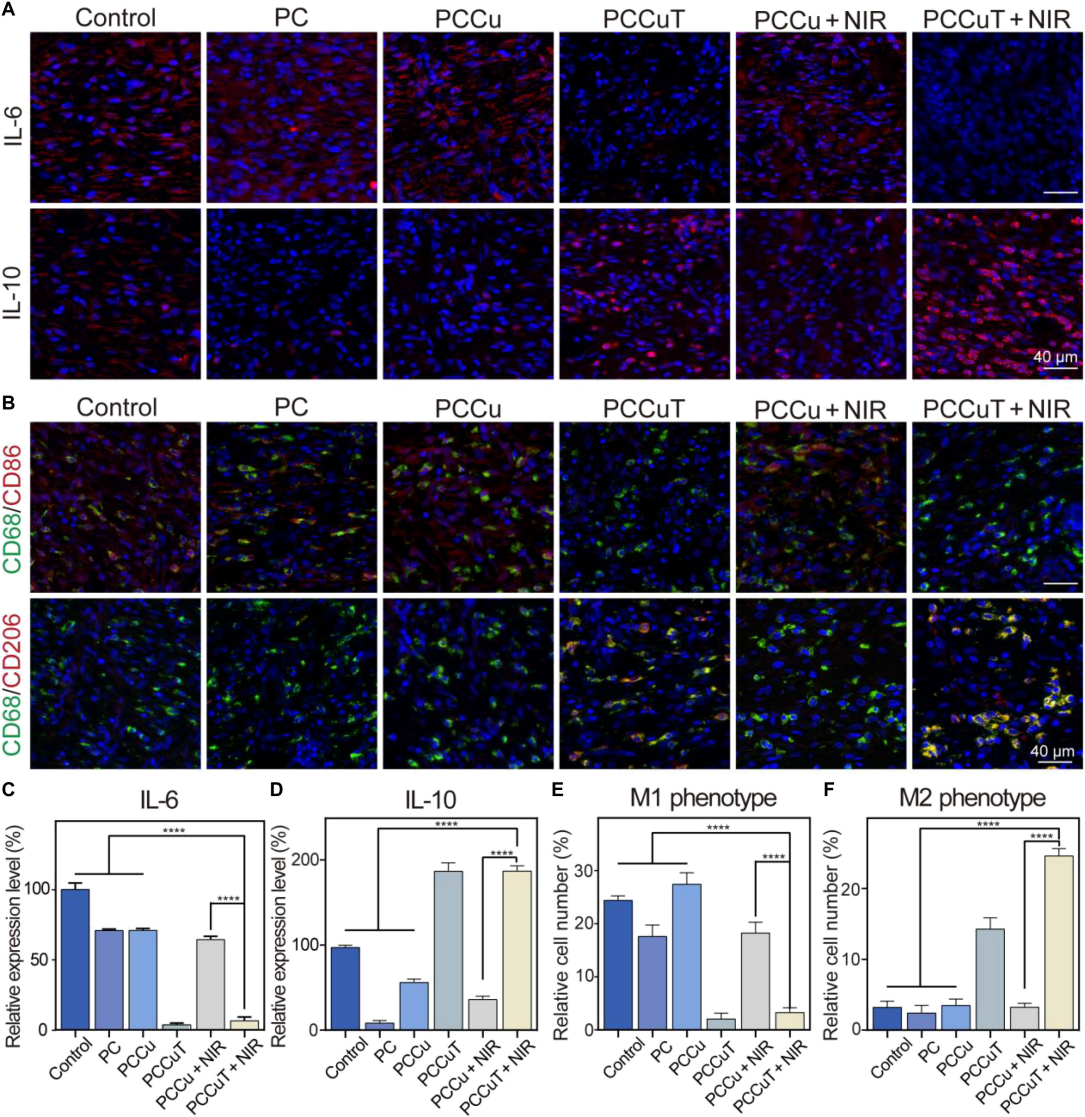
Analysis of wound regeneration tissue using immunofluorescence techniques. (A) Immunofluorescence staining for interleukin-6 (IL-6) and interleukin-10 (IL-10) in skin tissues. (B) Immunofluorescence staining by CD68-marked (green) macrophages, followed by CD86-marked (red) M1-type macrophages and CD206-marked (red) M2-type macrophages. Relative expression levels of (C) IL-6 and (D) IL-10 and the number of (E) M1-type macrophages and (F) M2-type macrophages.

To assess the anti-inflammatory effects of the PCCuT hydrogel in vivo, we performed immunofluorescence staining for IL-6 and IL-10 in periwound tissues. Previous research indicates that IL-6 is released by immune cells during the early phases of infection, leading to heightened inflammatory responses [45]. Conversely, IL-10 is a cytokine known for its anti-inflammatory properties; it promotes cell growth and inhibits pro-inflammatory cytokines such as IL-6 and tumor necrosis factor alpha by activating macrophages [46]. As shown in Fig. 9A, IL-6 levels were significantly elevated in the control, PC, PCCu, and PCCu + NIR groups compared to those in the PCCuT and PCCuT + NIR groups, which exhibited notably higher IL-10 expression (Fig. 9C and D). This effect is attributed to the TA-Fe complex’s capacity to suppress inflammation, lowering IL-6 levels while enhancing IL-10 production.

Macrophages are essential in the environment of wound healing. In wounds resulting from diabetes, the shift from M1 to M2 macrophages is frequently disrupted, obstructing the repair mechanisms. In our research, we marked macrophages using CD68 (green) and distinguished between M1 and M2 types through CD86 (red) and CD206 (red), respectively. As depicted in Fig. 9B, M1 macrophages were prevalent in the control, PC, PCCu, and PCCu + NIR groups, while the PCCuT and PCCuT + NIR groups showed a marked reduction in M1 cells and an increase in M2 macrophages (Fig. 9E and F). This shift is largely due to the regulatory influence of the TA-Fe MPN in promoting M2 differentiation. Overall, these findings indicate that the PCCuT hydrogel effectively reduces pro-inflammatory factor secretion, increases anti-inflammatory factor expression, and facilitates macrophage polarization toward the M2 phenotype.

**Fig. 10.**
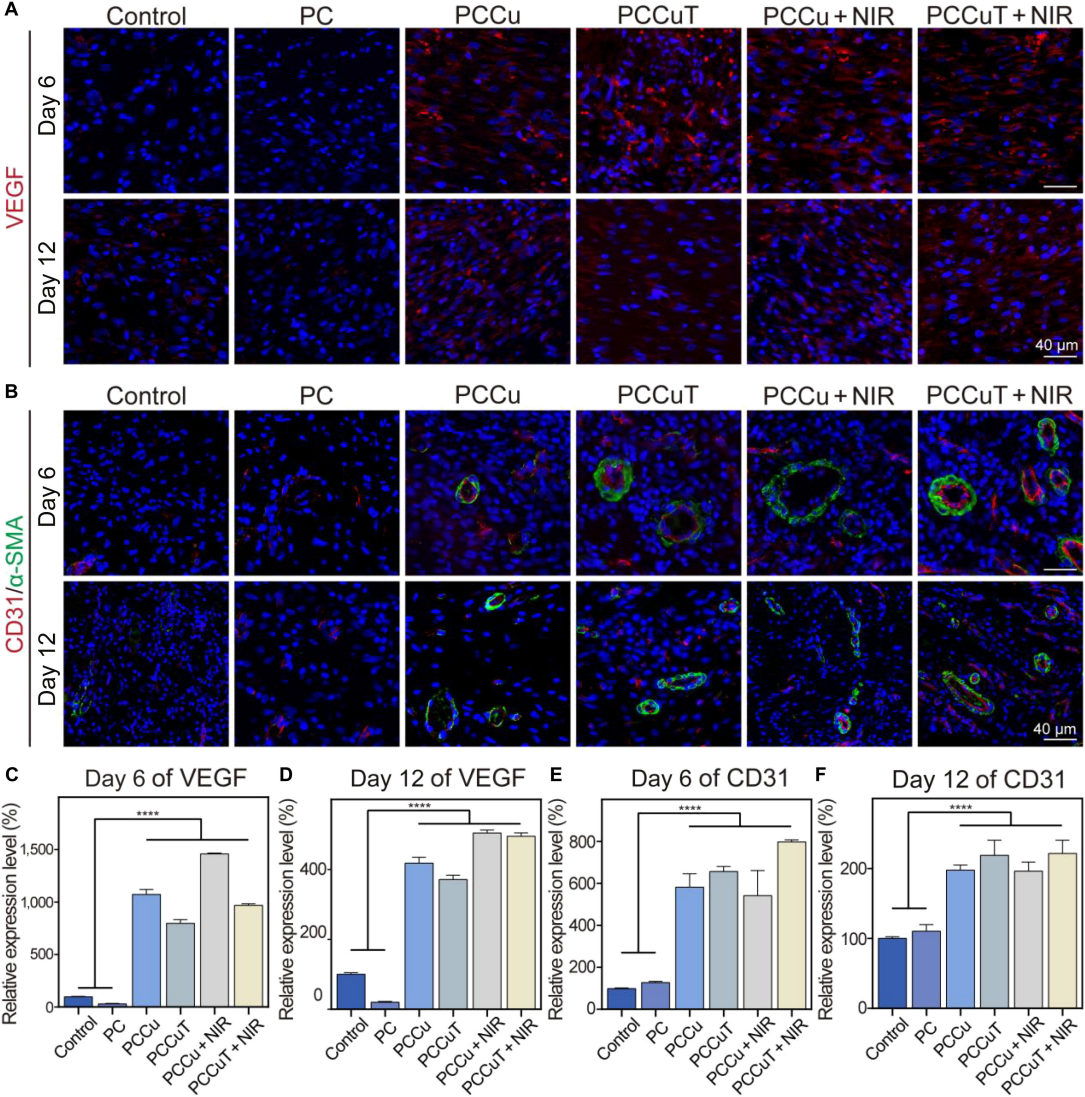
Immunofluorescence staining of wound regeneration tissue. (A and B) Immunofluorescence staining of vascular endothelial growth factor (VEGF), CD31, and alpha-smooth muscle actin (α-SMA) in wound tissues on days 6 and 12. The relative expression level of VEGF on (C) day 6 and (D) day 12. The relative expression level of CD31 on (E) day 6 and (F) day 12.

An immunofluorescence assay was performed to label VEGF, CD31 (which is a marker for neovascular endothelium), and α-SMA (indicative of smooth muscle cells) to evaluate the angiogenic capabilities of the PCCuT hydrogel. VEGF is essential in the later phases of inflammation and tissue healing, as it is important for granulation tissue formation, vascular proliferation, and collagen production [47]. As shown in Fig. 10A, the levels of VEGF in the PCCu, PCCuT, PCCu + NIR, and PCCuT + NIR groups were significantly higher than those in the PC group at both 6 and 12 d (Fig. 10C and D), likely due to the angiogenic properties of elemental copper.

CD31, a transmembrane protein found in neovasculature, serves as an early angiogenesis biomarker [48]. Similarly, α-SMA is considered a smooth muscle actin marker. In Fig. 10B, CD31 expression was notably higher in the PCCu, PCCuT, PCCu + NIR, and PCCuT + NIR groups compared to that in the control and PC groups on day 6 (Fig. 10E). On day 12, elevated CD31 levels were also observed in the copper-containing groups, correlating with the angiogenic effects attributed to copper ions (Fig. 10F). The PCCu + NIR treatment strategy significantly alleviated bacterial infection at the wound site, with the secretion of copper ions increasing the expression of VEGF, CD31, and α-SMA, which contributed to improving the microenvironment of diabetic wounds. In contrast, the PCCuT + NIR treatment further addressed oxidative stress, promoted M2 macrophage polarization, and facilitated effective wound healing.

## Conclusion

In summary, CuS-Se NPs were synthesized for the first time, and CuS-Se@TA-Fe NPs were obtained by functionalizing TA-Fe MPN on their surfaces. CuS-Se@TA-Fe NPs were further loaded into PVA/CMCS hydrogel to obtain a composite hydrogel (PCCuT). The PCCuT hydrogel demonstrates excellent self-healing and adhesion properties. Furthermore, the PCCuT composite hydrogel exhibits nanoenzymatic effects similar to those of SOD and CAT, which are capable of scavenging excessive reactive ROS and generating oxygen. In addition, the PCCuT composite hydrogel has a significant photothermal effect, which can effectively eliminate bacteria in the wound. Experiments conducted both in vitro and in vivo provided evidence that the composite hydrogel had notably decreased the expression levels of inflammatory factors, promoted angiogenesis, facilitated the polarization of M2-type macrophages, and effectively promoted the healing of diabetic infected wounds. Collectively, the PCCuT hydrogel is a promising wound dressing for the treatment of diabetic infected wounds.

## Data Availability

The data that support the findings of this study are available from the corresponding authors upon reasonable request.
