# Erratum to “From Hemostasis to Angiogenesis: A Self-Healing Hydrogel Loaded with Copper Sulfide-Based Nanoenzyme for Whole-Process Management of Diabetic Wounds”

**DOI:** 10.34133/bmr.0240

**Published:** 2025-08-08

**Authors:** Chuankai Zhang, Peirong Zhou, Shoucheng Li, Xuancheng Zhang, Zhaoxin Xia, Zihan Rao, Xuemin Ma, Yajuan Hu, Yongcen Chen, Junliang Chen, Yun He, Gang Tao, Rui Cai

**Affiliations:** ^1^ Luzhou Key Laboratory of Oral & Maxillofacial Reconstruction and Regeneration, The Affiliated Stomatological Hospital, Southwest Medical University, Luzhou 646000, China.; ^2^ Department of Oral and Maxillofacial Surgery, The Affiliated Stomatological Hospital, Southwest Medical University, Luzhou 646000, China.; ^3^ Institute of Stomatology, Southwest Medical University, Luzhou 646000, China.; ^4^ Department ofGeneral Dentistry, The Affiliated Hospital, Southwest Medical University, Luzhou 646000, China.; ^5^ Department of Oral and Maxillofacial Surgery, The Affiliated Hospital, Southwest Medical University, Luzhou 646000, China.

In the Research Article “From Hemostasis to Angiogenesis: A Self-Healing Hydrogel Loaded with Copper Sulfide-Based Nanoenzyme for Whole-Process Management of Diabetic Wounds”, the authors made an error in Figure 6B [1]. While organizing the data from the antimicrobial experiments, it was discovered that the raw data from one of the earlier test batch experiments had been mistakenly entered into the images of the current paper. In the image of the experiment against E. coli in Figure 6B, the image of the PCCuT+NIR plate was attached to the image of the other batch, which was an oversight in the organization of the data. We sincerely regret this error and deeply apologize for any inconvenience caused to the editorial team and readers.

**Figure 6.**
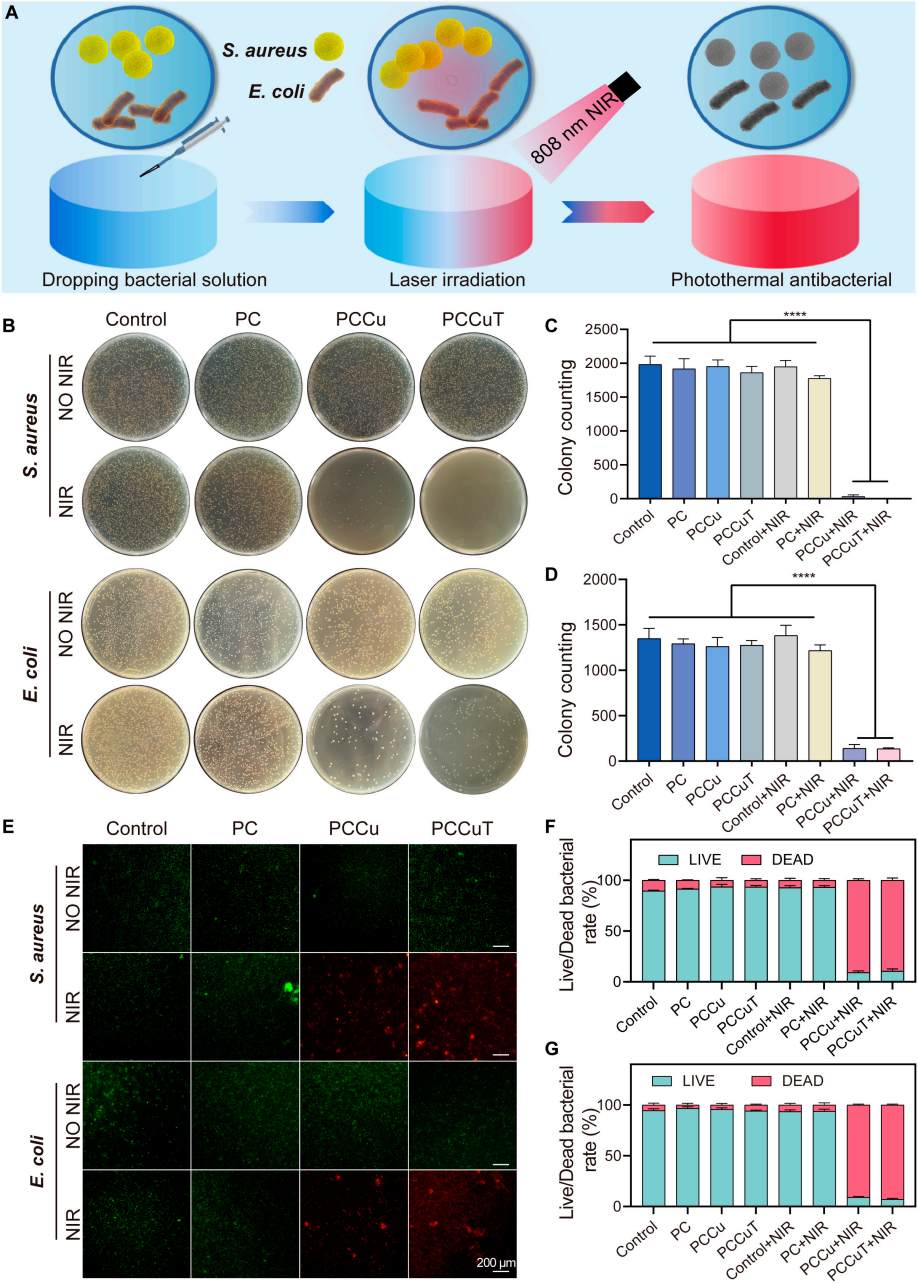
In vitro antimicrobial properties of PC, PCCu, and PCCuT hydrogels. (A) Schematic representation of the hydrogel’s photothermal antibacterial ability. (B) Images of colony formation after near-infrared (NIR; 808 nm, 0.8 W/cm^2^) irradiation of PC, PCCu, and PCCuT hydrogels. (C) Statistics of *Staphylococcus aureus* colony counts. (D) Statistics of *Escherichia coli* colony counts. (E) Live/dead stained images of *S. aureus* and *E. coli*. Live/dead bacterial rate of (F) *S. aureus* and (G) *E. coli*.

Figure 6B has now been corrected in the PDF and HTML, and it is also presented below.
