# Supplementary material for: From Hemostasis to Angiogenesis: A Self-Healing Hydrogel Loaded with Copper Sulfide-Based Nanoenzyme for Whole-Process Management of Diabetic Wounds
Source: Biomater Res. 2025 May 23;29:0208. doi: 10.34133/bmr.0208 (PMC12099055; doi:10.34133/bmr.0208)
Supplement: Supplementary 1 — Figs. S1 to S17 [file bmr.0208.f1.doc]

**Supporting Information**

**
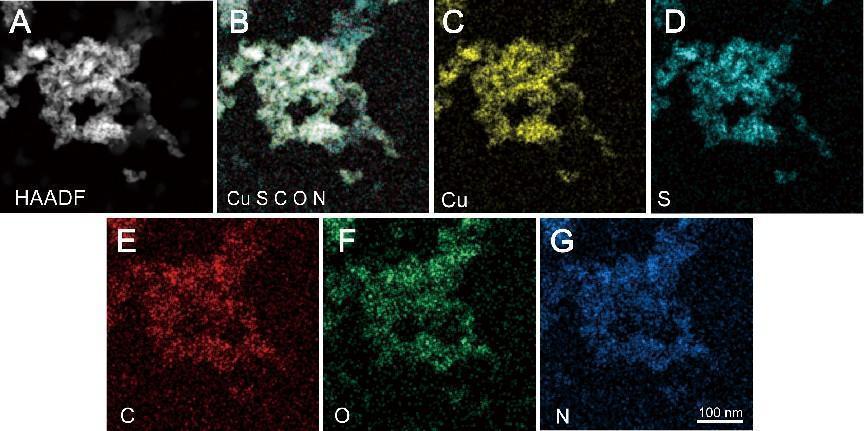
**

Fig. S1. Elemental mapping of CuS-Se NPs.


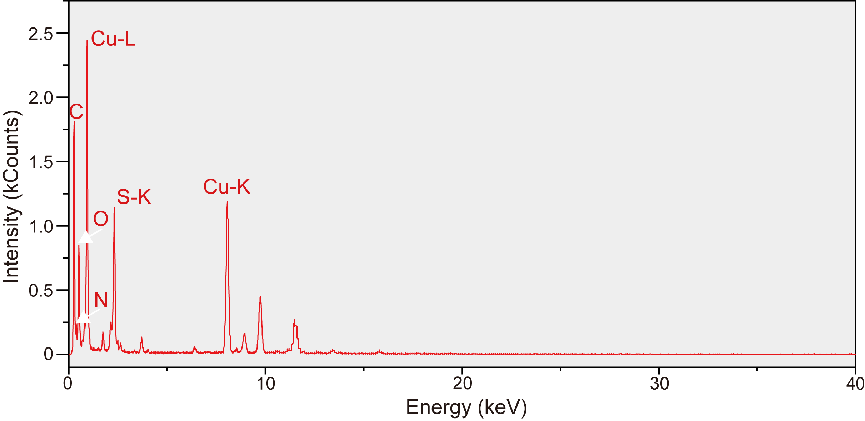


Fig. S2. EDS of CuS-Se NPs


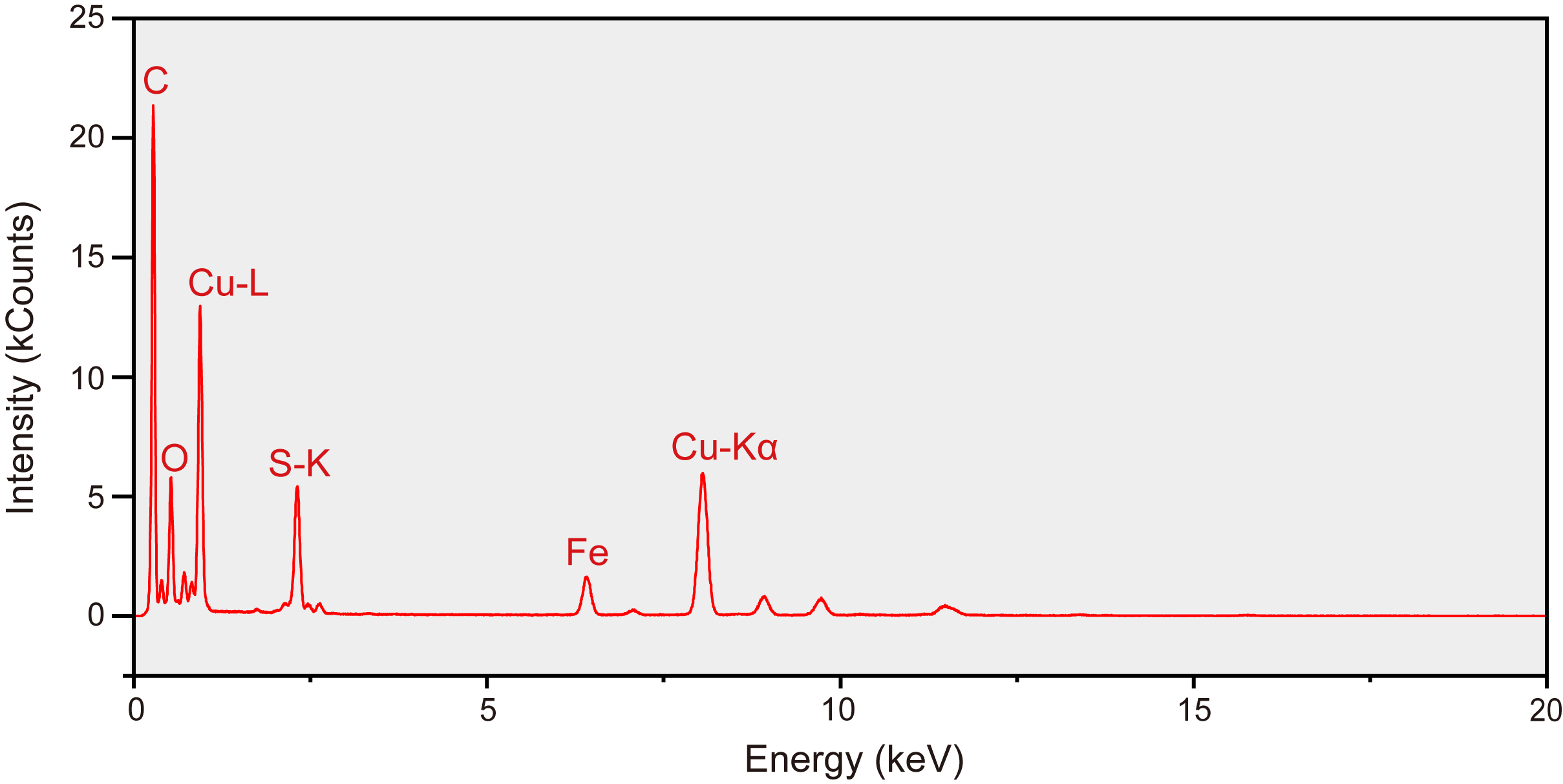


Fig. S3. EDS of CuS-Se@TA-Fe NPs.


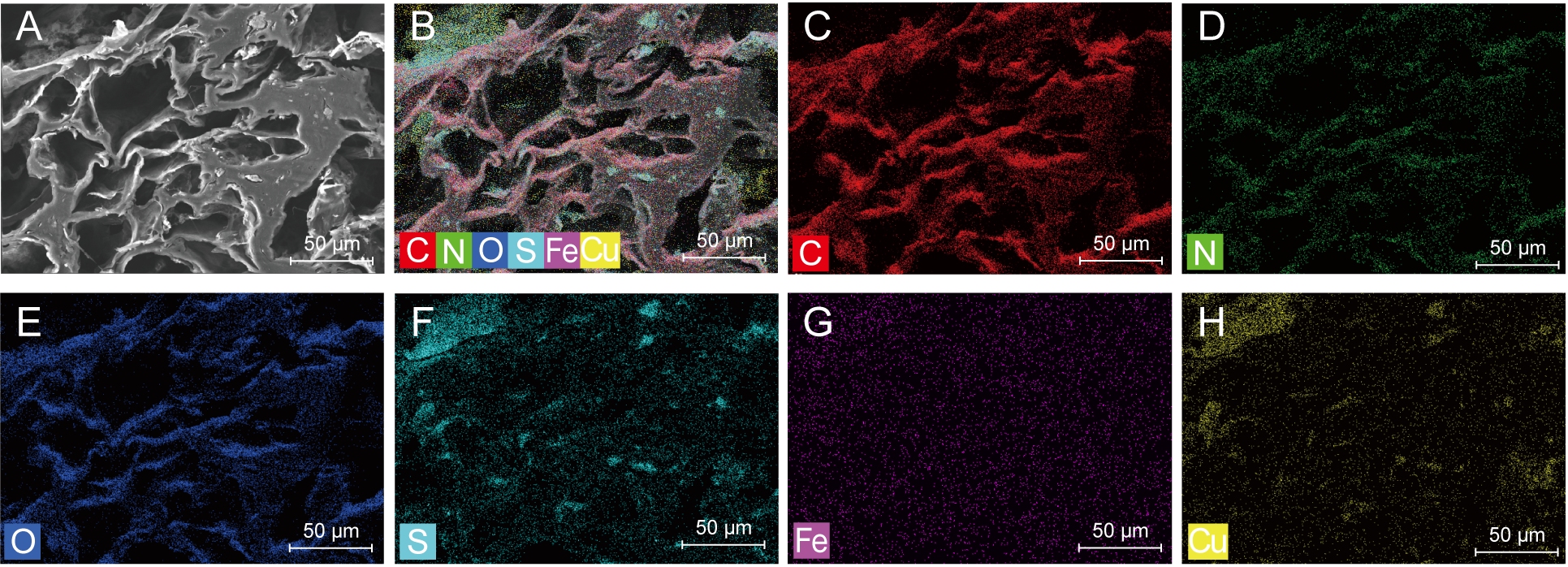


Fig. S4. Morphological and elemental characterization of PCCuT hydrogel. (A) TEM image of PCCuT hydrogel. (B) HAADF-STEM image of PCCuT hydrogel. (C-H) Elemental mapping of C, N, O, S, Fe, and Cu.


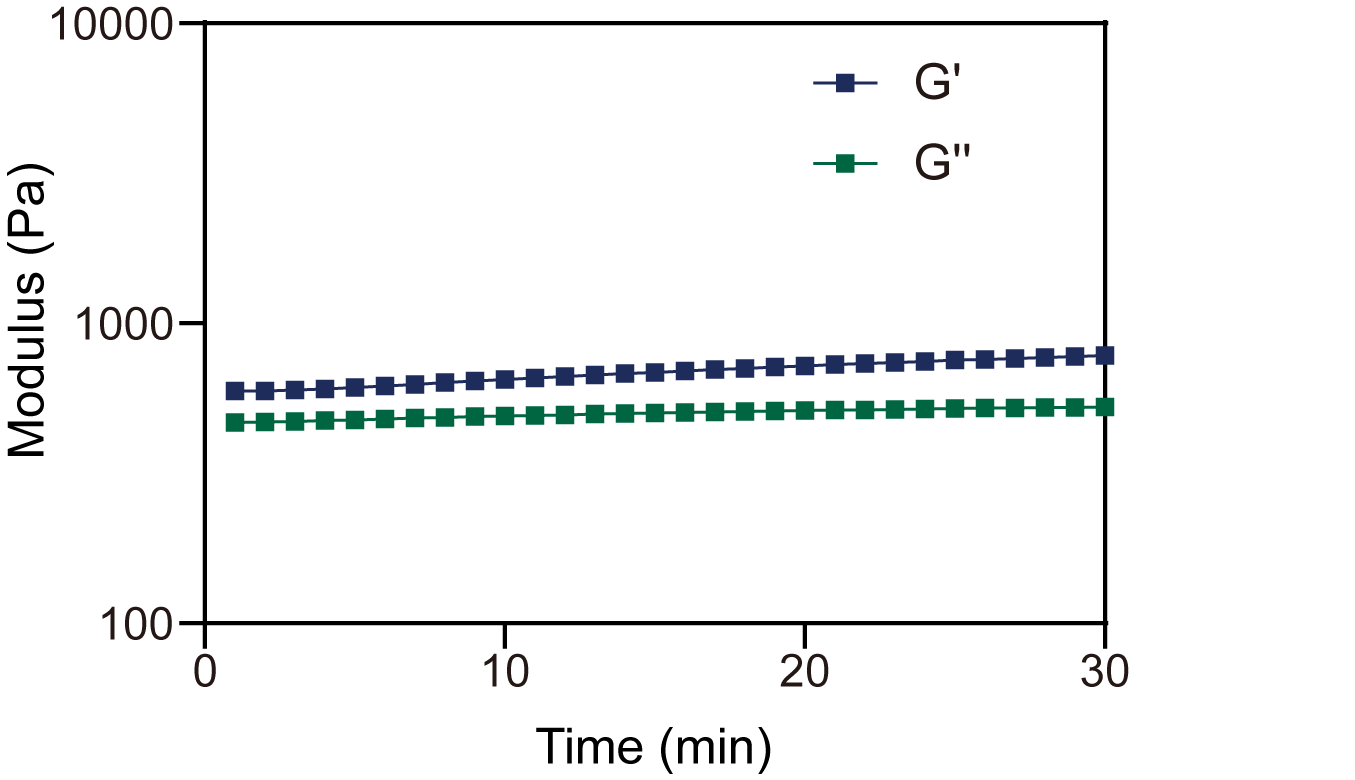


Fig. S5. The rheological curve of the PCCuT hydrogel under time scan mode.


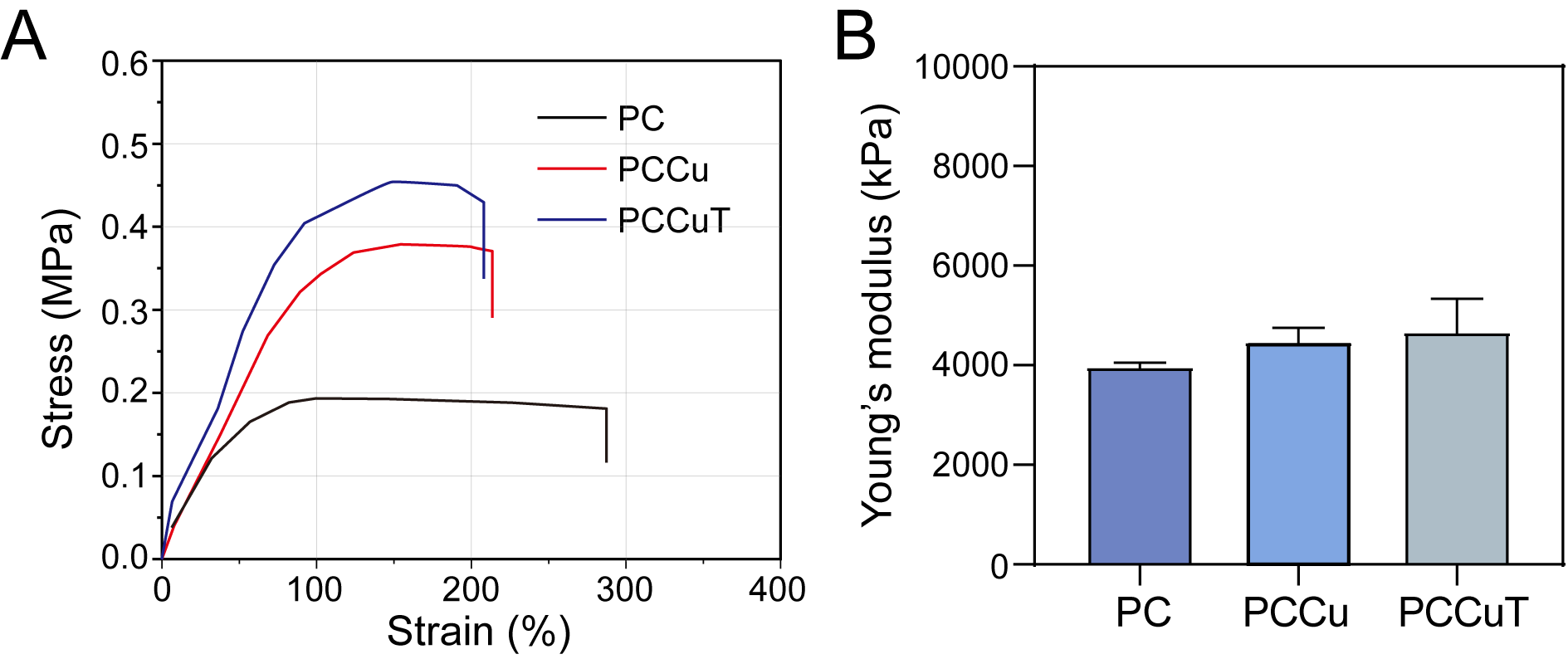


Fig. S6. Mechanical properties of hydrogels. (A) Stress-strain curves of the hydrogels. (B) Young's modulus of the hydrogels.


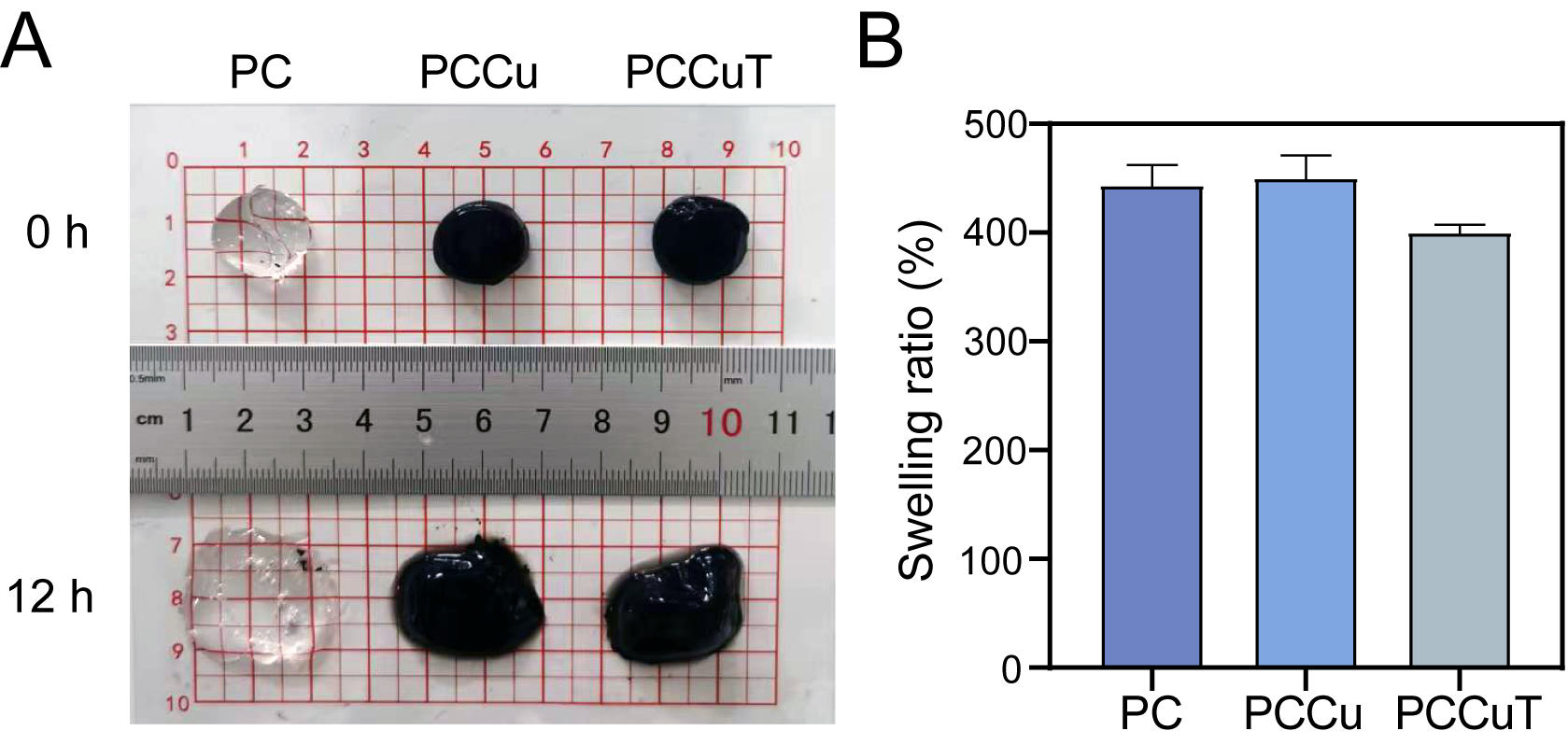


Fig. S7. (A) Swelling behavior of different hydrogels after soaking in PBS for 12 h. (B) Swelling ratios of hydrogels in different groups.


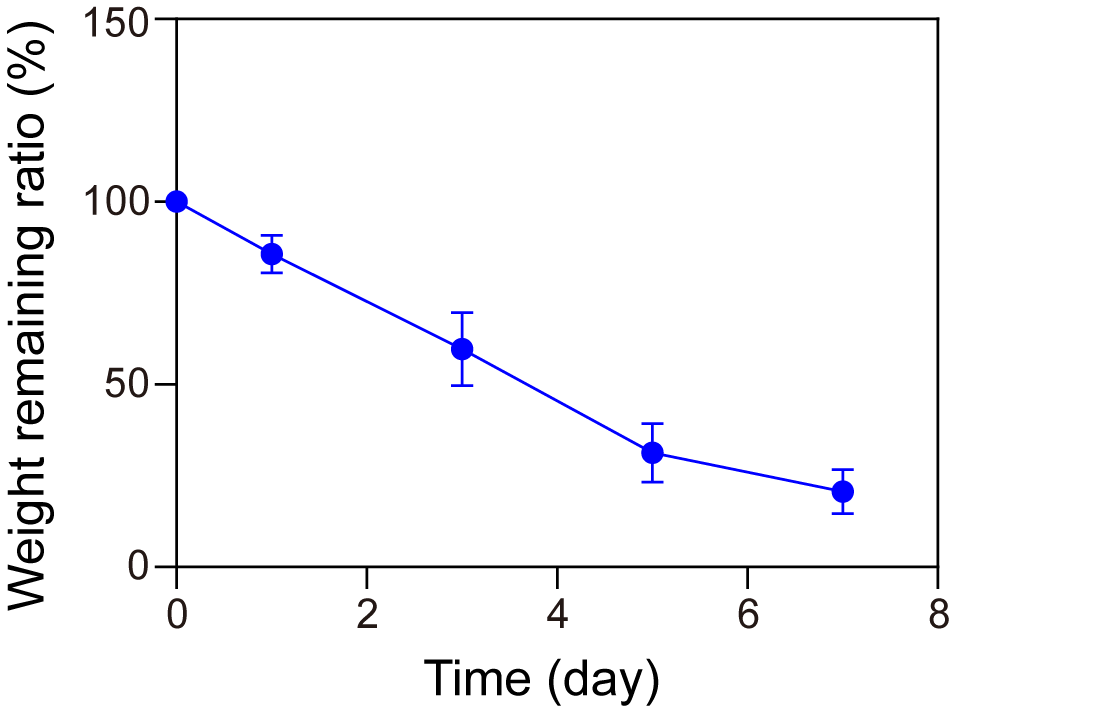


Fig. S8. The weight remaining ratio of PCCuT hydrogel in SBF.


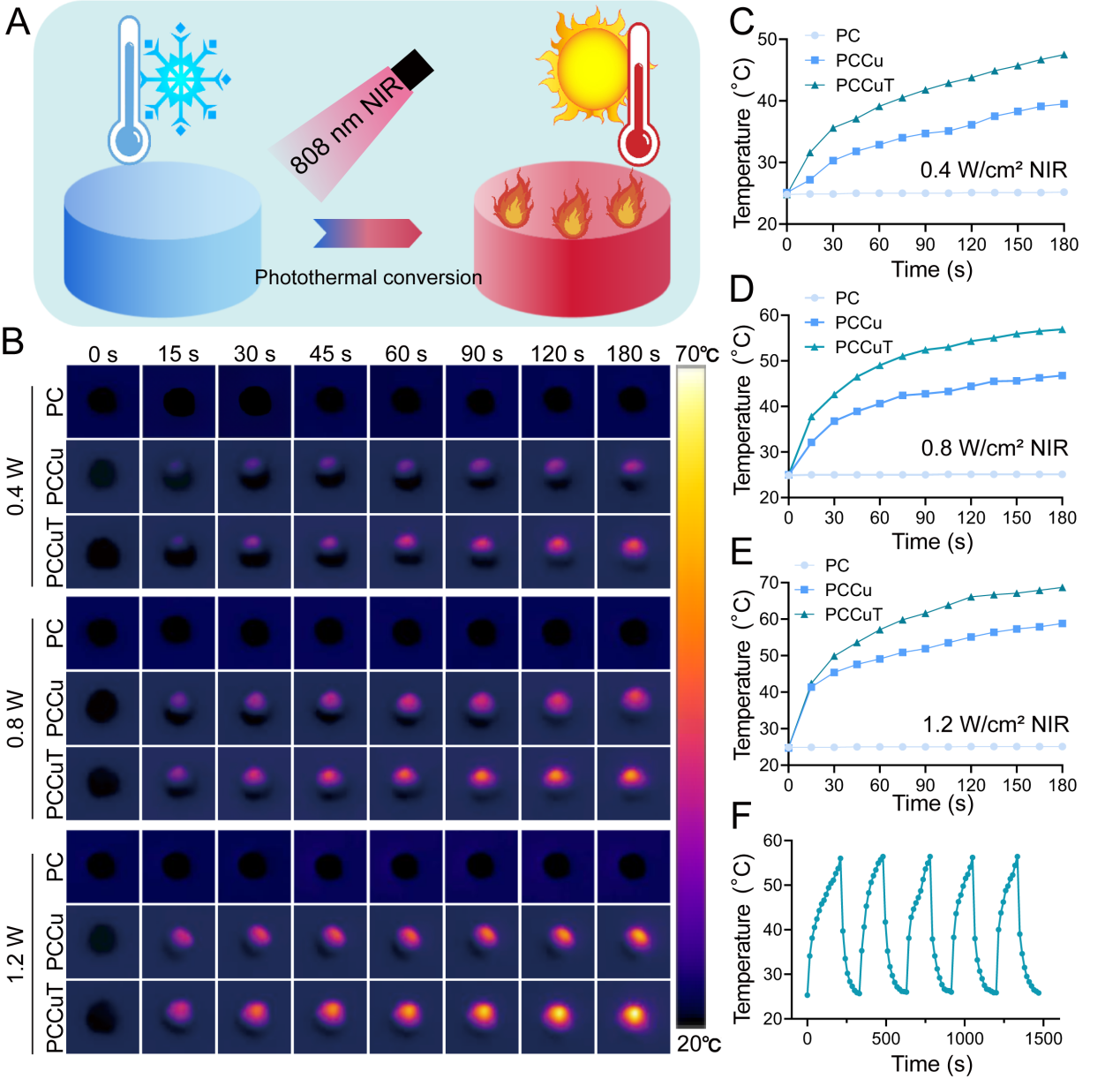


Fig. S9. Photothermal properties of PC, PCCu, and PCCuT hydrogels. (A) Schematic diagram showing the photothermal conversion property of hydrogels. (B) Real-time infrared thermogram of hydrogel under 808 nm NIR for 180 s. (C-E) Photothermal effect curves of three kinds of hydrogels under infrared illumination at different wattages. (F) Switching cycle experiment under NIR irradiation.


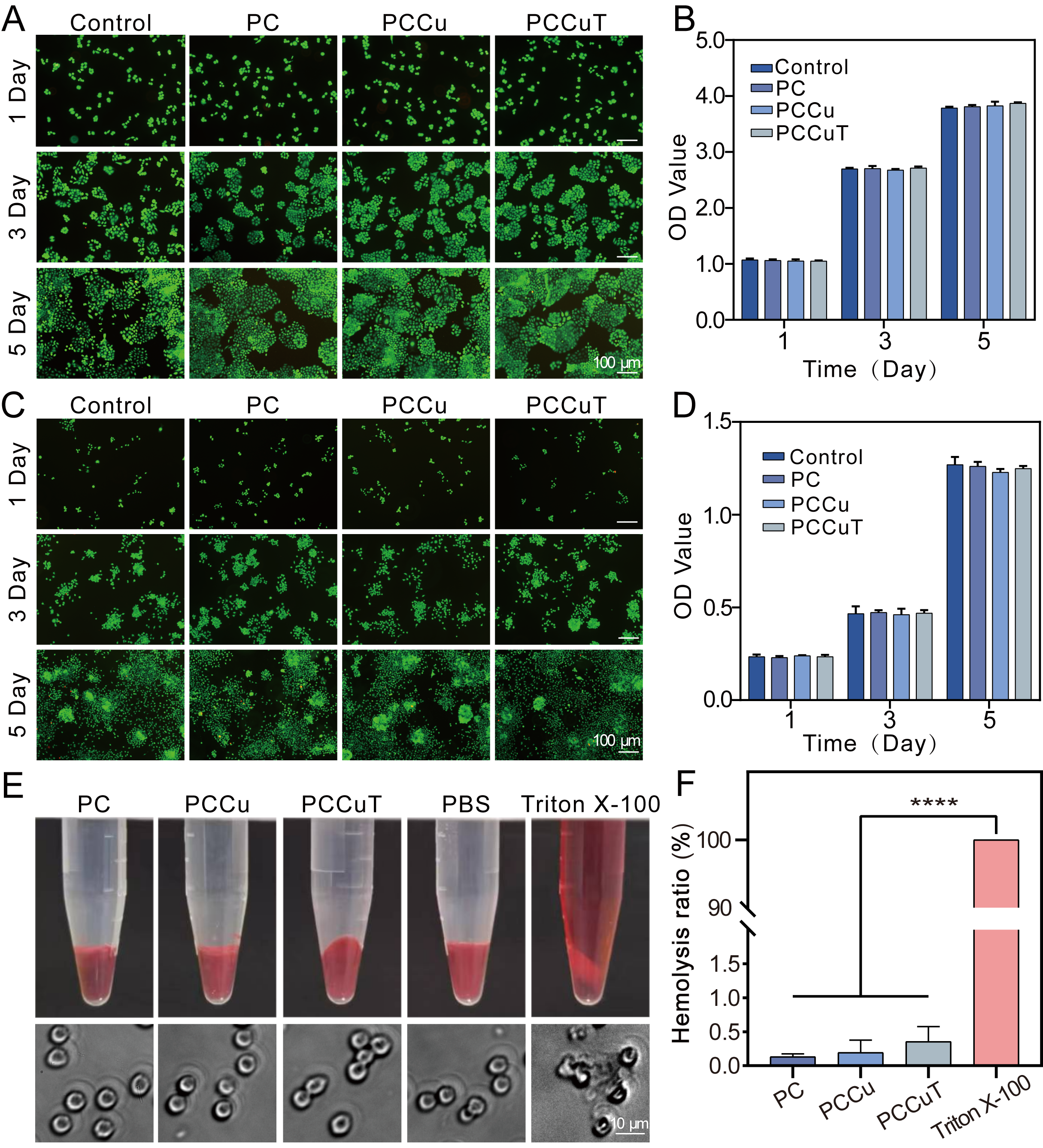


Fig. S10. *In vitro* hemocompatibility and cytocompatibility assays. (A) Live/dead fluorescence images and (B) cell viability of HUVECs cells after hydrogel treatment for 1, 3, and 5 days. (C) Live/dead fluorescence images and (D) cell viability of RAW264.7 cells after hydrogel treatment for 1, 3, and 5 days. (E) Hemolysis images and microscopic morphology of erythrocytes after composite hydrogel incubation. (F) Hemolysis rate.


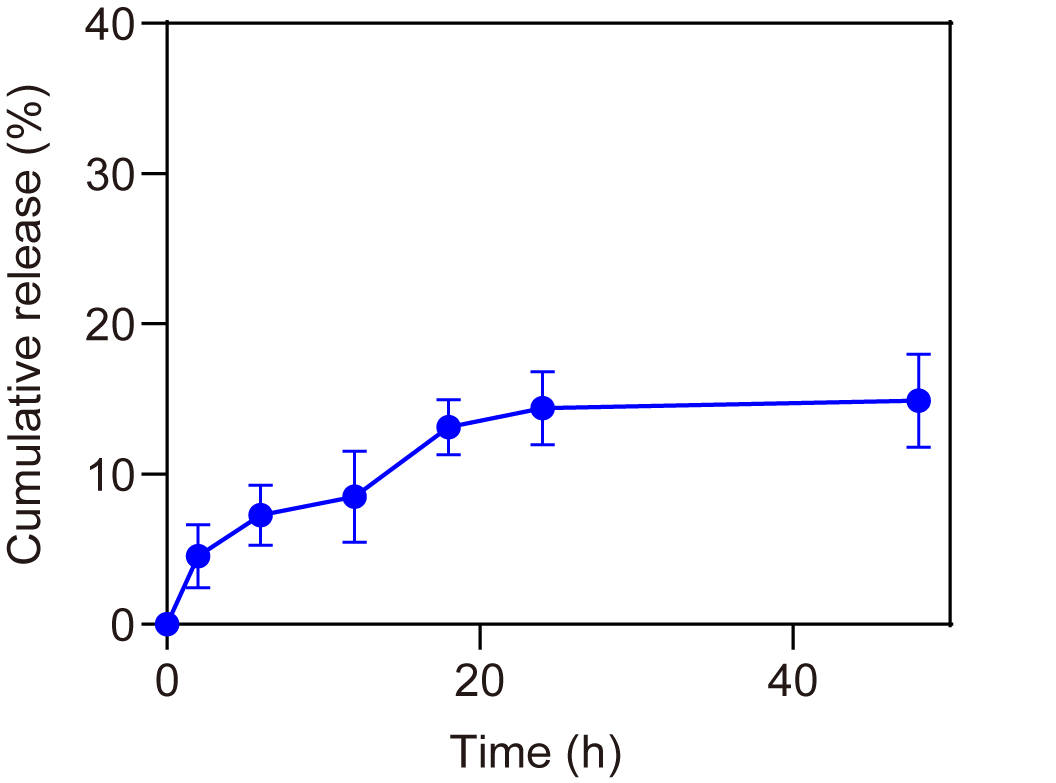


Fig. S11. Cumulative release profiles of Cu^2+^ in 48 h.


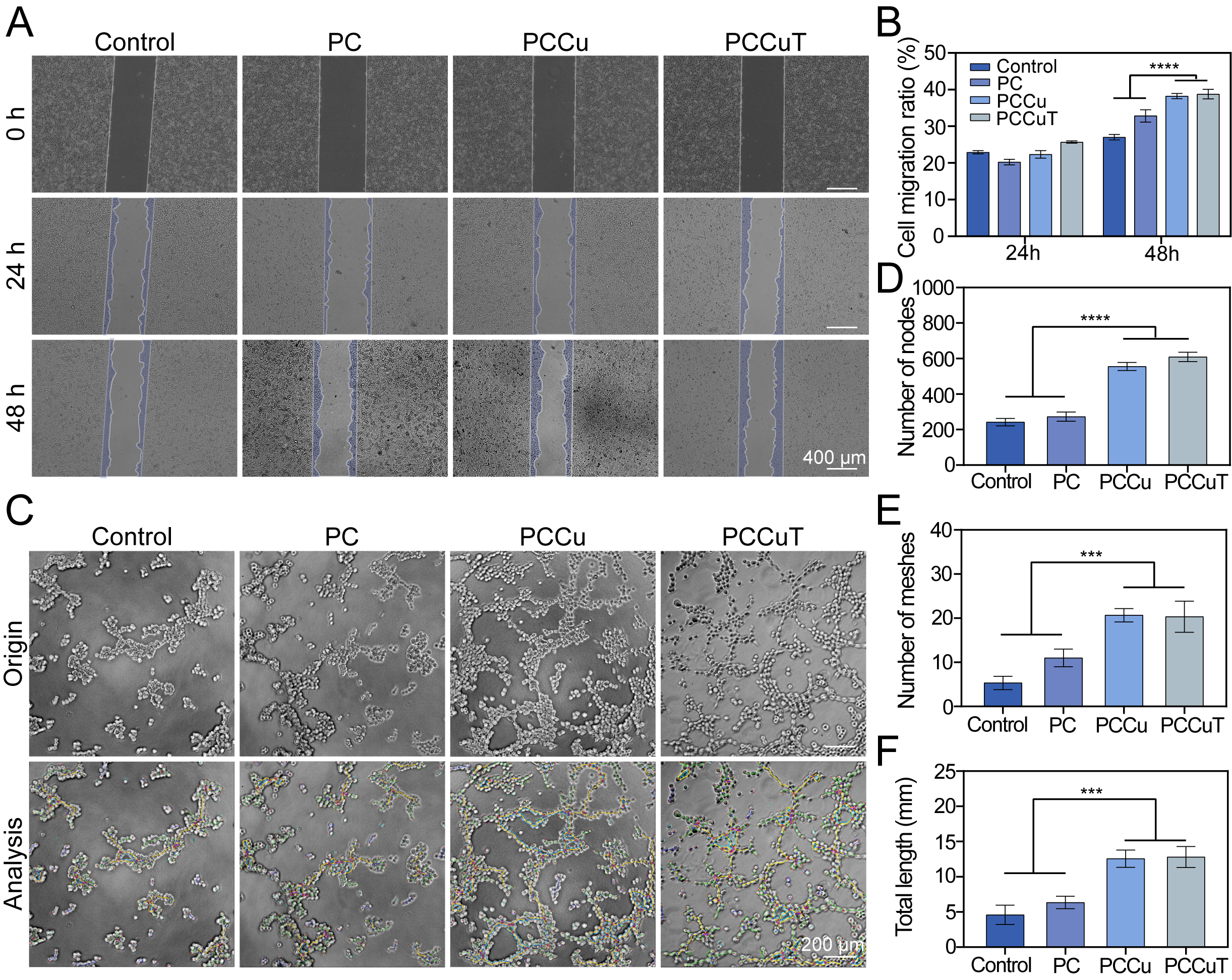


Fig. S12. Evaluation of PCCuT hydrogel *in vitro* for enhancing endothelial cell migration and angiogenesis. (A) Representative images of the scratch assay. (B) Analysis of cell migration ratio in scratch assay. (C) Representative images of tube-forming experiments. (D) Quantitative analysis of the number of nodes, (E) the number of meshes, and (F) the total length of tube formation in the tube formation experiment.


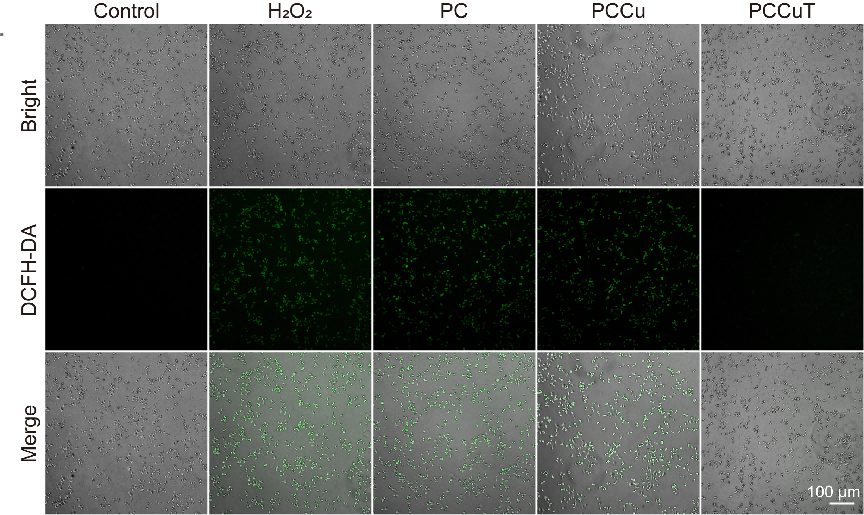


Fig. S13. Detection of intracellular ROS levels by DCFH-DA fluorescent probe.


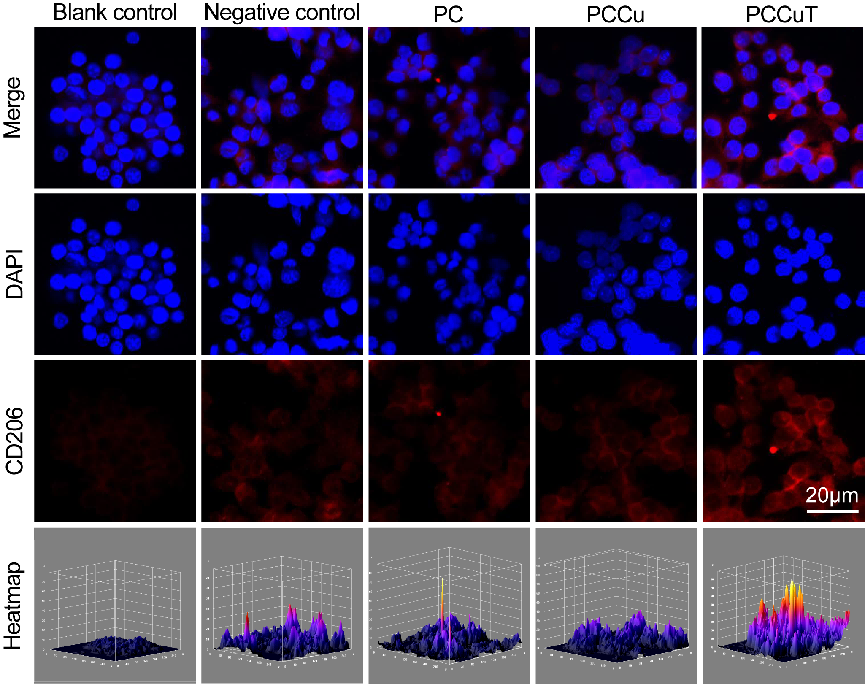


Fig. S14. Immunofluorescence staining images of M2-type macrophages (CD206).


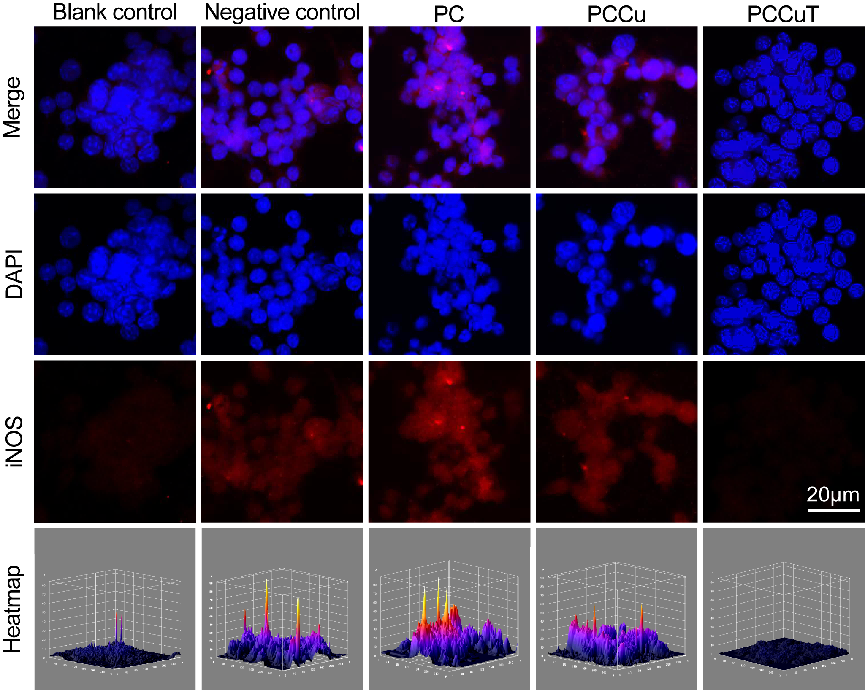


Fig. S15. Immunofluorescence staining images of M1-type macrophages (iNOS).


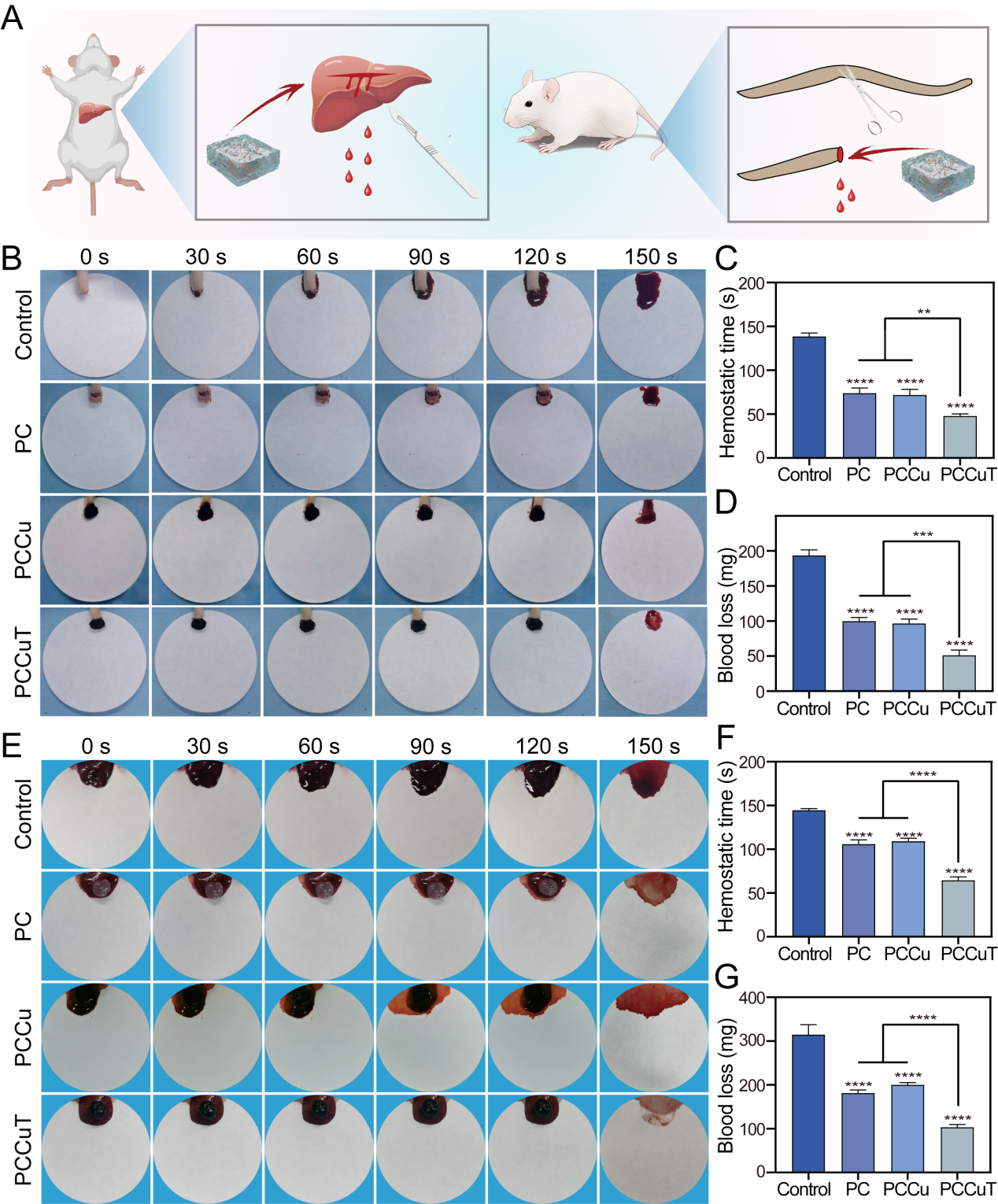


Fig. S16. Evaluating the hemostatic properties of PCCuT hydrogel. (A) Schematic diagram of hemostatic treatment of PCCuT hydrogel in rat liver and tail. (B) Pictures of composite hydrogels for the treatment of tail injuries in rats. (C) Hemostasis time and (D) bleeding weight of the rat tail trauma model. (E) Picture of composite hydrogel treatment of rat liver trauma. (F) Hemostasis time and (G) bleeding weight of the rat liver trauma model.


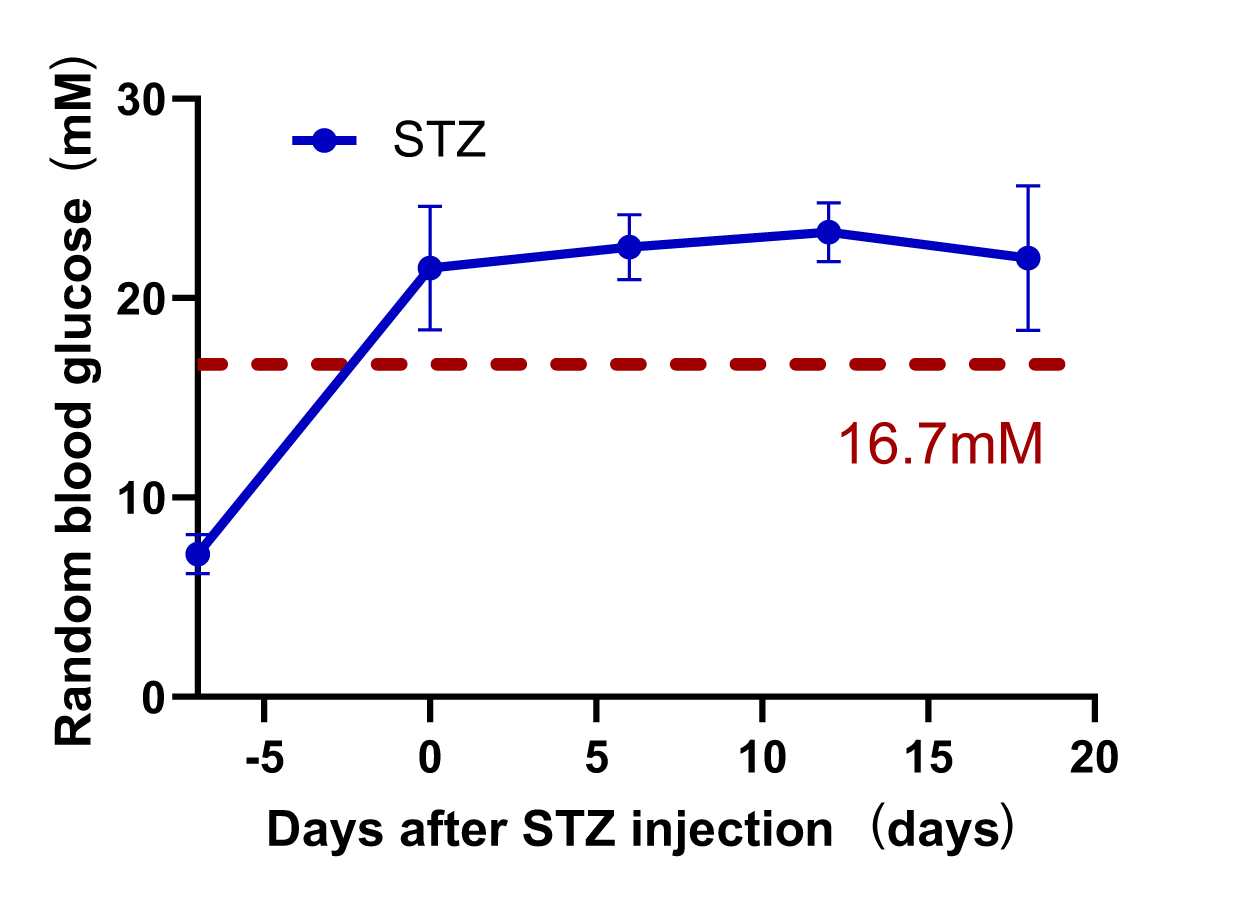


Fig. S17. Detection of blood glucose level in diabetic rats.
